# Cardiovascular Effectiveness and Safety of Antidiabetic Drugs in Patients with Type 2 Diabetes and Peripheral Artery Disease: Systematic Review

**DOI:** 10.3390/medicina60091542

**Published:** 2024-09-20

**Authors:** Antonio Cimellaro, Michela Cavallo, Marialaura Mungo, Edoardo Suraci, Francesco Spagnolo, Desirée Addesi, Medea Pintaudi, Carmelo Pintaudi

**Affiliations:** 1 Internal Medicine Unit, Department of Medicine Specialties, “Pugliese-Ciaccio” Hospital of Catanzaro, Azienda Ospedaliero-Universitaria Renato Dulbecco, Via Pio X n.83, 88100 Catanzaro, Italy; miche.cavallo@gmail.com (M.C.); edoardosuraci88@gmail.com (E.S.); francescospagnolomd@gmail.com (F.S.); desi.addesi@libero.it (D.A.); c.pintaudi@libero.it (C.P.); 2 Internal Medicine Unit, Department of Medical and Surgical Sciences, ‘Magna Græcia’ University of Catanzaro, Viale Europa, Località Germaneto, 88100 Catanzaro, Italy; marialaura.mungo@gmail.com; 3 Unit of Plastic Surgery, Department of Surgery, Azienda Ospedaliero-Universitaria “Gaetano Martino”, 98124 Messina, Italy; pintaudi.medea@gmail.com

**Keywords:** peripheral artery disease, diabetes, cardiovascular events, MACE, MALE, lower-limb amputation, lower extremities complications, glucose-lowering drugs

## Abstract

Peripheral artery disease (PAD) is an atherosclerotic condition commonly complicating type 2 diabetes (T2D), leading to poor quality of life and increased risk of major adverse lower-limb (MALE) and cardiovascular (CV) events (MACE). Therapeutic management of PAD in T2D patients is much more arduous, often due to bilateral, multi-vessel, and distal vascular involvement, in addition to increased systemic polyvascular atherosclerotic burden. On the other hand, the pathophysiological link between PAD and T2D is very complex, involving mechanisms such as endothelial dysfunction and increased subclinical inflammation in addition to chronic hyperglycemia. Therefore, the clinical approach should not ignore vascular protection with the aim of reducing limb and overall CV events besides a mere glucose-lowering effect. However, the choice of the best medications in this setting is challenging due to low-grade evidence or lacking targeted studies in PAD patients. The present review highlighted the strong relationship between T2D and PAD, focusing on the best treatment strategy to reduce CV risk and prevent PAD occurrence and worsening in patients with T2D. The Medline databases were searched for studies including T2D and PAD up to June 2024 and reporting the CV effectiveness and safety of the most used glucose-lowering agents, with no restriction on PAD definition, study design, or country. The main outcomes considered were MACE—including nonfatal acute myocardial infarction, nonfatal stroke, and CV death—and MALE—defined as lower-limb complications, amputations, or need for revascularization. To the best of our current knowledge, GLP-1 receptor agonists and SGLT2 inhibitors represent the best choice to reduce CV risk in T2D and PAD settings, but a personalized approach should be considered. GLP-1 receptor agonists should be preferred in subjects with prevalent atherosclerotic burden and a history of previous MALE, while SGLT2 inhibitors should be used in those with heart failure if overall CV benefits outweigh the risk of lower-limb complications.

## 1. Introduction

The global prevalence of diabetes mellitus has increased continuously, affecting almost half a billion people worldwide, with an estimated prevalence rising by more than 50% in the next 25 years [1]. Of interest, patients with type 2 diabetes (T2D) have an average reduction in life expectancy of 5–10 years and a poor quality of life because of an increased risk of cardiovascular (CV) disease [2,3]. The close relationship between T2D and CV disease can be attributed to a pathophysiological common soil, characterized mostly by insulin resistance with chronic sustained hyperinsulinemia, hyperglycemia, and advanced glycation end-products (AGEs), abnormal redox status, endothelial dysfunction with increased risk of vascular damage, and atherosclerosis [4,5,6,7]. The presence of such mechanisms explains why T2D is often associated with other CV risk factors, such as obesity, hypertension, and dyslipidemia, which interplay together to lead to poor outcomes [8,9]. On the other hand, the occurrence of CV complications significantly impacts costs, accounting for 5–11% of total healthcare spending in European countries [10] and amounting to approximately EUR 336 and 8409 per person per year, respectively, for uncomplicated and complicated diabetes [11]. With the aim of reducing costs, ameliorating the quality of life, and improving the prognosis of subjects affected by T2D, treatment strategy to prevent CV complications plays a pivotal role [12]. On this basis, the United States Food and Drug Administration (FDA) revised industry rules to mandate CV risk assessment for new diabetic drugs through rigorous and targeted randomized controlled trials [13]. Despite promising results on CV risk provided by new therapies, some uncertainty remains in specific settings, such as the subjects affected by T2D and peripheral arterial disease (PAD), which represents one of the most important vascular complications with elevated morbidity and mortality [14,15,16]. The present review highlighted the strong relationship between T2D and peripheral artery disease (PAD), overviewing dynamics and trends, understanding related pathophysiological mechanisms, and focusing on the best treatment strategy to reduce CV risk and prevent PAD occurrence and worsening in patients with T2D, to the best of our current knowledge and evidence.

## 2. Epidemiological and Pathophysiological Link between T2D and PAD

PAD is an atherosclerotic condition characterized by chronic occlusion of the arteries in the lower extremities, but it also represents a marker of generalized systemic atherosclerosis and is associated with elevated CV morbidity and mortality [14,15,16]. Diabetes is a well-known, strong, and independent risk factor for vascular damage, increasing the risk of PAD from 1.89 to 4.05 in comparison to subjects without [17]. According to different diagnostic modalities and clinical settings, the published prevalence rates of PAD in diabetes vary widely between studies, from 8 to 30% [18] to about 22% in patients with newly diagnosed T2D [19], until 50% in diabetic patients affected by foot ulcers [18]. The research into gender differences has documented that PAD is less frequent in women than in age-matched men; however, women are more likely to have atypical symptoms and greater walking impairment with progressive functional decline [20]. Notably, PAD progresses more rapidly and leads to worse outcomes in diabetic than nondiabetic patients, resulting in a higher risk of functional impairment, mobility loss, infectious complications, amputations, and CV mortality [21,22,23,24,25,26]. In comparison with nondiabetics, diabetic patients with PAD are generally younger, have a higher body mass index and much more CV comorbidities, and are more often suffering from neuropathic symptoms and polyvascular disease [27,28,29]. In addition, PAD in patients with diabetes has a number of characteristics that render it more difficult to treat, such as diffuse arterial wall calcification, multi-level and multi-vessel involvement, bilateral and mainly distal obstruction, long and extensive narrowing plaques [30,31,32]. Furthermore, it has been demonstrated that the recruitment of collateral circulations and the neoangiogenesis process, an expected adaptive and compensatory response to the reduced flow in obstructive arterial disease, is reduced in diabetic subjects [33].

Regarding pathophysiological issues, mechanisms underlying the interaction between T2D and PAD are multiple and do not only concern poor glycemic control. Certainly, chronic hyperglycemia induces vascular damage, as demonstrated by the research of Selvin and coworkers, in which every 1% increase in hemoglobin A1c contributed to a 26–28% increased risk of PAD [34]. Indeed, it is well-established that chronic hyperglycemia is associated with non-enzymatically modification and accumulation of abnormal proteins and lipids, known as AGEs, which are involved in vascular stiffness and atherosclerotic plaque instability [35,36]. Accumulating evidence suggests that AGEs are independent predictors of PAD, consolidating the need to improve glycemic control to prevent vascular damage in diabetic patients [35,36]. Beyond glycemic control, it has been demonstrated that insulin resistance is independently associated with vascular damage, with a risk of PAD almost doubled also after adjustment for confounding factors [37]. In addition, diabetic patients with elevated C-reactive protein have a 2-times higher risk of PAD when compared with those without, suggesting an important role of chronic subclinical inflammation that usually characterizes cardio-metabolic conditions [38]. Taken together, these data suggest that T2D and PAD are strongly and complexly interrelated, making treatment strategy much more arduous due to the need to see beyond mere glycemic control.

## 3. Materials and Methods

The current systematic review adheres to the predetermined protocol of the Preferred Reporting Items for Systematic Reviews and Meta-Analyses (PRISMA) guidelines [39]. Since this article is based on previously conducted studies and does not contain any new studies with human participants or animals performed by any of the authors, ethical implications were not deemed relevant to the drafting of this review. These authors have conducted a comprehensive systematic search of Medline (PubMed) from its inception until 6 June 2024. The study keywords included “type 2 diabetes” and “peripheral artery disease” or “peripheral arterial disease”, associated with “cardiovascular” or “vascular” or “lower limb” or “lower extremities” or “amputation”, combined with the various pharmacological classes (“metformin”, “GLP-1 receptor agonists”, “DPP-4 inhibitors”, “SGLT2 inhibitors”, “glinides”) or the single drugs [“metformin”; “liraglutide”, “semaglutide”, “exenatide”, “dulaglutide”, “albiglutide”, “lixisenatide”; “alogliptin”, “linagliptin”, “saxagliptin”, “sitagliptin”, “vildagliptin”; “empagliflozin”, “canagliflozin”, “dapagliflozin”, “sotagliflozin”, “ertugliflozin”; “tirzepatide”; “acarbose”; “repaglinide”) available for prescription in T2D patients, along with the Boolean operators AND and OR. Additional searches were conducted through references cited in the included studies to ensure comprehensive coverage.

Regarding the eligibility criteria to be included in the review, studies needed to measure or focus on a specific topic: CV effectiveness and safety of approved glucose-lowering agents in T2D, with special attention on the PAD setting. The main inclusion criteria for relevant publications were as follows: (1) adult patients with T2D; (2) any clinical study design; (3) all comparators; (4) epidemiology, experimental, and clinical data; (5) studies published up to June 2024; (6) any countries; (7) studies written in English. Of importance, these authors focused on the PAD setting, considering targeted studies if published; if specifically designed studies were not available, subanalysis on subgroups affected by PAD was considered. No restrictions on PAD definition or diagnostic modalities were applied among inclusion criteria. Preference was expressed for meta-analyses, randomized clinical trials, guidelines, and systematic reviews. Therefore, in addition to or in case of the absence of the previous ones, prospective and retrospective studies were considered if relevant to the topic. The authors have also included studies on combination therapy between two or more agents, as usual in clinical practice, specifying when for each drug in the appropriate drug sections. The main outcomes considered were composite major adverse CV events (MACE) and major adverse limb events (MALE). The first included nonfatal acute myocardial infarction, nonfatal stroke, and CV death; the second included lower-limb complications, amputations, or the need for revascularization. If different outcomes were considered relevant to the topic, they were specified in the drug sections as appropriate. If available, experimental in vitro and in vivo research were also included if they were considered pertinent and showed molecular and pathophysiological mechanisms about CV implications by the glucose-lowering agents, with the aim to support and explain clinical findings observed in clinical studies or to bridge the literature gap. Papers were excluded if they did not fit into the conceptual framework of the study, were based on people aged <18 years old, were designed for type 1 diabetes, were unrelated to T2D and PAD or vascular damage, followed patients for less than one months, or were written in languages other than English. A summary of the main evidence cited in this review, providing for each research study design, inclusion/exclusion criteria, mean age, gender, mean follow-up, main CV, and limb outcomes, was reported in Table S1 as Supplementary Material.

The literature search, data extraction and interpretation, quality assessment, and judgment were independently and in pairs performed by two authors with experience and expertise [AC and MC]. The authors first screened the titles and the abstracts, focusing on the relevance of the topic and then using the pre-defined eligibility criteria; if deemed relevant to the topic, the full-text articles were reviewed. Regarding data extraction and interpretation, any disagreements were resolved through consensus and consultation of a third senior author [CP] if further clarification was needed. A flow diagram of the selection process is reported in Figure S1 as Supplementary Material.

## 4. Evidence on CV Effectiveness and Safety of Antidiabetic Drugs in Patients with T2D and PAD

### 4.1. Metformin

Over the last 60 years, metformin has been the most commonly used glucose-lowering agent, becoming the first-line medication for newly diagnosed T2D in most international guidelines [40]. In clinical practice, metformin improves glycemic management with a significant reduction in glycated hemoglobin of up to 2.0% [41], similar to sulfonylureas, thiazolidinediones and glucagon-like peptide-1 receptor agonists (GLP-1 RA) in head-to-head trials [41,42] and, in general, showing greater effectiveness than dipeptidyl peptidase 4 (DPP-4) inhibitors and sodium-glucose transport protein 2 inhibitors (SGLT2i) [42,43]. Despite its widespread use, to date, its therapeutic mechanisms are complex and not yet fully clarified. Mainly, metformin exerts its prevailing action on cell’s energy metabolism, with a glucose-lowering effect by inhibiting hepatic gluconeogenesis and, to a lesser effect, by enhancing insulin-mediated glucose uptake in peripheral tissue [44]. On the vascular side, it has been proven that metformin can improve endothelial function, intimal thickening, vascular calcification, and inflammation in both in vitro [45,46] and in vivo models [47]. With regard to CV and non-CV outcomes, in a substudy of the United Kingdom Prospective Diabetes Study (UKPDS) on overweight patients, those on metformin experienced, respectively, 42% and 36% fewer diabetes-related and all-cause deaths when compared to diet alone arm, and a lower mortality rate than sulfonylureas or insulin arms with an overall risk reduction for myocardial infarction of 33% after a 10-year follow-up [48,49]. In the ADOPT trial (A Diabetes Outcome Prevention Trial), CV events were low overall, but no differences between groups (metformin vs. rosiglitazone vs. glyburide) were assessed [50]. More recent data regarding 34,000 subjects showed that the assumption of metformin was associated with a lower incidence of CV death and all-cause mortality (HR 0.8; *p* < 0.001) [51]. Of interest, the REACH (Reduction of Atherothrombosis for Continued Health) register focused on outcomes in diabetics with known atherothrombosis. Among over 19,000 patients, metformin was associated with a low rate of CV death and all-cause mortality, especially in those with heart failure, renal damage, or older than 65 years [52]. Moreover, in a meta-analysis involving 1,066,408 diabetics affected by coronary artery disease, the group on metformin had lower all-cause mortality (HR 0.67) and, in particular, lower CV mortality (HR 0.81) and incidence of new CV events (HR 0.83) [53]. As a result, in a recent era in which large CV outcome trials are required and showed net benefit by administration of SGLT2i or GLP-1 RA, metformin could maintain a pivotal role in CV prevention. Indeed, over two thirds of patients enrolled in EMPA-REG [54], LEADER [55], and CANVAS [56] had metformin at baseline with no data available about head-to-head comparison. Furthermore, in a pooled analysis of DPP-4 inhibitors, there were differences between DDP-4 inhibitors alone or in combination with metformin, with a trend toward improved CV outcomes for the latter (HR 0.92; 95% CI 0.84–1.01) [57]. With concern to PAD, Tan and coworkers found a protective effect of metformin on MALE also in a multivariable analysis (adjusted OR 0.26; 95% CI 0.10–0.68) in a prospective study on 100 patients with T2D and PAD [58]. A recent retrospective analysis assessed the question of whether metformin could affect the outcome of endovascular and open surgical intervention in diabetics [59]. The authors enrolled 1204 consecutive patients who underwent endovascular or open revascularization for chronic limb ischemia, paying attention to patency, limb salvage, major adverse limb events (MALE), major adverse cardiac events (MACE), and survival rates. After a mean follow-up of 48 months, after adjustment for other risk factors and when compared to insulin and other oral antidiabetics, metformin was associated with improved survival and decreased MACE in patients affected by PAD who underwent open or endovascular intervention, similar to nondiabetic patients. On the other hand, there were no differences in patency or limb salvage rates between metformin and other hypoglycemics. In another retrospective analysis, Kibrik et al. tested the hypothesis that metformin could decrease restenosis after an endovascular procedure for PAD [60]. In this cohort of 187 patients, after a follow-up of 13 months, there were no statistically significant differences between groups (diabetics on metformin vs. diabetic on other hypoglycemic vs. nondiabetics) about limb loss, restenosis, or re-intervention rates. Finally, another study is ongoing and will evaluate the effect of metformin on functional status, PAD progression, systemic inflammation, and CV events in nondiabetic patients with intermittent claudication [61]. Based on the above statement, there is much evidence to consolidate the administration of metformin to improve CV outcomes in diabetic patients, with some studies supporting its prescription also with the aim to prevent the progression of vascular damage and MALE in patients with PAD at early stages. However, in diabetics with chronic limb ischemia who have undergone open or endovascular intervention, metformin seems to be able to improve survival and reduce MACE after revascularization but not significantly ameliorate restenosis, patency, and limb salvage rates in comparison to other antidiabetic drugs.

### 4.2. Sulfonylureas

Sulfonylureas, including glyburide, glipizide, and glimepiride, are a low-cost class with long clinical experience and established glycemic efficacy by enhancing insulin secretion. Despite that, current guidelines suggest against the use of sulfonylureas in favor of novel drugs due to a large body of evidence demonstrating adverse effects such as hypoglycemia and weight gain, as well as long-standing uncertainty regarding CV safety [62]. The safety concern on CV risk associated with sulfonylureas depends on high rates of CV mortality attributed directly to a possible cardiotoxic effect by interaction with cardiac potassium channels and indirectly by hypoglycemia [63]. Despite some bias, some observational studies and meta-analyses supported this concern since sulfonylurea users had a higher risk of myocardial infarction, all-cause, and CV mortality than other antihyperglycemic drugs [64]. Regarding the PAD setting, the safety of sulfonylureas was investigated only as a comparator, showing non-superiority and often inferiority in comparison with other drugs in terms of lower-limb amputations [65]. Taken together and considering the growing evidence supporting novel glucose-lowering drugs, sulfonylureas should not be considered a main treatment in patients with T2D and PAD.

### 4.3. Thiazolidinediones

Pioglitazone and rosiglitazone belong to the class of peroxisome proliferator-activated receptor agonists, also known as glitazones or thiazolidinediones, with the ability to improve insulin-sensitivity and reduce hyperglycemia, in addition to promising vascular effect resulting from experimental studies [66]. Of interest, pioglitazone significantly reduced arterial stiffness in comparison with insulin and sulfonylureas in a prospective, nonrandomized, open-label trial [67] and slowed the progression of coronary atherosclerosis in patients with coronary artery disease in comparison with sulfonylureas [68]. With the aim to assess the CV effect of pioglitazone, The PROspective pioglitAzone Clinical Trial In macroVascular Events (PROactive) was achieved. Over 5000 patients with T2D and previous CV macrovascular events were enrolled, randomly assigned to pioglitazone or placebo plus conventional therapy, and followed for a mean of 34.5 months. The primary endpoint was the composite of all-cause mortality, nonfatal myocardial infarction, stroke, acute coronary syndrome, endovascular or surgical intervention in the coronary or leg arteries, and amputation above the ankle; the main secondary endpoint was the composite of all-cause mortality, nonfatal myocardial infarction, and stroke. Despite a neutral effect on the primary endpoint (HR 0.90, 95% CI 0.80–1.02; *p* = 0.095), pioglitazone reached the secondary endpoint (HR 0.84, 95% CI 0.72–0.98; *p* = 0.027), demonstrating CV benefit in T2D patients with high CV risk [69]. Regarding PAD, in a post hoc analysis of the PROactive study, patients with PAD at baseline showed significantly higher rates of the composite primary endpoint (CV event or death), all-cause mortality, and stroke than those with no PAD. The beneficial CV effect of pioglitazone observed in the overall population was not confirmed in patients with peripheral atherosclerosis; of interest, there was a higher rate of leg revascularizations in the pioglitazone group, especially in the first 12 months of treatment. Indeed, among the overall PROactive population, patients without PAD seemed to benefit more from pioglitazone treatment than those with PAD [70]. Furthermore, safety concerns about a reported increased risk of hospitalization for heart failure with rosiglitazone have questioned the real and net CV benefit of thiazolidinediones [71]. In accordance with these data, it is possible to speculate that glitazones are an interesting glucose-lowering class with pleiotropic, vascular, and anti-inflammatory effects but with a non-sufficiently demonstrated CV net benefit to recommend the administration in the setting of PAD.

### 4.4. GLP-1 RA

As part of the incretin family, GLP-1 is a polypeptide hormone secreted from the upper gastrointestinal tract in response to food consumption, with the ability to increase insulin secretion by binding to pancreatic islet B receptors in a glucose-dependent manner. Additional effects of GLP-1 RA include suppressing appetite with premature satiety and decreasing post-meal glycemia by delayed gastric emptying [72]. Due to its properties and the ability to reduce glycated hemoglobin by 1–2%, GLP-1 has become important in the treatment of T2D as an analogous drug with heterogeneous pharmacokinetic patterns [73]. Of interest, not all tested GLP-1 RA were capable of reducing CV events with such differences in CV effects between the agents. For example, liraglutide and semaglutide had structural homology to native GLP-1 and showed more CV effects than the exendin-4-based agents lixisenatide and exenatide. The main evidence regarding GLP-1 RA and CV effects, with special attention on the PAD setting where data were available, is reported below.

#### 4.4.1. Liraglutide

Liraglutide is a long-acting GLP-1 RA, produced by recombinant DNA and administered by daily subcutaneous injections, as it has poor bioavailability when administered orally [72]. In addition to beneficial effects on body weight, blood pressure, glycemic and lipid profile that endorsed its use in obesity and T2D, it has been demonstrated that liraglutide significantly reduced CV risk [74]. On the bench side, it has been proven that liraglutide reduced oxidative stress, apoptosis, and microvascular damage and counteracted endothelial dysfunction and inflammation in vascular and endothelial cells [75,76]. In a clinical setting, the effects of liraglutide on CV effectiveness and safety have been demonstrated by the LEADER study conducted on 9340 high-CV-risk patients with a positive history of previous CV events [55]. After a median follow-up of 3.8 years, the primary composite endpoint characterized by the first occurrence of death from CV causes, nonfatal myocardial infarction, or nonfatal stroke, occurred in significantly fewer patients in the liraglutide group than in the placebo group (13% vs. 14.9%; HR 0.87; 95% CI 0.78–0.97; *p* < 0.001 for noninferiority; *p* = 0.01 for superiority). Furthermore, fewer patients died from both CV and any causes in the liraglutide group (respectively, 4.7% vs. 6.0% with HR 0.78, *p* = 0.007; 8.2% vs. 9.6% with HR 0.85, *p* = 0.02). The frequencies of nonfatal myocardial infarction and nonfatal stroke were lower in the liraglutide group, although the differences were not significant. Despite gastrointestinal side effects as the main reason for discontinuation, the incidence of severe adverse events was not significantly different between the liraglutide and placebo groups. Of interest, liraglutide reduced the onset of MACE both in patients with PAD (15.5% in the liraglutide group vs. 19.6% in the placebo group; HR 0.77, 95% CI 0.58–1.01) and in those without PAD history (12.7% vs. 14.1%; HR 0.89, 95% CI 0.79–1.00; *p* = 0.34). Further confirmations of CV benefits in PAD setting derive from post hoc analysis of the LEADER trial: Dhatariya et al. have reported that liraglutide led to a reduced risk of amputations in comparison with placebo (HR 0.65, 95% CI 0.45–0.95; *p* = 0.03), despite similar frequencies of diabetes-related foot ulcers events between groups (3.8% [176/4668] versus 4.1% [191/4672], respectively; HR 0.92, 95% CI 0.75–1.13; *p* = 0.41]) [77]. To assess more specifically the effect of liraglutide on PAD, the recent STARDUST study was conducted [78]. In this randomized open-label clinical trial, 55 patients with T2D and PAD were enrolled between February 2021 and June 2022, with a final follow-up in December 2022, and randomized to receive 1.8 mg of subcutaneous liraglutide or conventional treatment for CV risk factors (control group with conventional glucose-lowering, lipid-lowering, antihypertensive, and antiplatelet or anticoagulant medications) for 6 months. The primary outcomes of the study were the modification from the baseline of peripheral perfusion and the comparison of the percentage of individuals who have achieved a 10% increase in the peripheral transcutaneous oxygen pressure (TcPo2) value compared to the basal in each group. The variation of TcPo2 was evaluated as the difference between the TcPo2 values measured at baseline and the end of the study. Secondary outcomes were glycemic and metabolic parameters (glycated hemoglobin, weight, body mass index, blood pressure, lipid profile, C-reactive protein, and renal function parameters. The STARDUST protocol also included the evaluation of a six-minute walking test (6MWT) as an exploratory result. The increase of at least 10% of TcPo2 compared to the baseline occurred in 24 participants (89%) belonging to the liraglutide group and 13 participants (46%) assigned to the control group (RR 1.91; 95% CI 1.26–2.90; *p* < 0.001). Moreover, TcPo2 appeared to increase over time in both the liraglutide and control groups, with significant differences in favor of the liraglutide group at the end of the study (estimated treatment difference, 11.2 mmHg; 95% CI 8.0–14.5 mmHg; *p* < 0.001). Furthermore, liraglutide was associated with a higher increase in circulating levels of endothelial progenitor cells and vascular endothelial growth factor-a compared to the control group at the end of the study. Regarding secondary outcomes, after 6 months, the liraglutide group showed a significant improvement in glycated hemoglobin, body mass index, blood pressure, C-reactive protein values, and distance on 6MWT. In summary, several pieces of evidence showed that liraglutide significantly reduced the overall incidence of MACE and mortality in T2D patients with high CV risk with a good safety profile. Among PAD patients, liraglutide maintains its effectiveness and is also able to prevent PAD progression and amputations, at least in part improving peripheral perfusion. These findings, supplemented by pleiotropic effects on the vascular system by ameliorating metabolic, hemodynamic, inflammatory, and functional patterns, make liraglutide a proven choice as medication for PAD in T2D.

#### 4.4.2. Semaglutide

Semaglutide is a GLP-1 RA with an extended half-life of approximately one week, which permits once-weekly subcutaneous administration [79]. Recently, an oral formulation with once-daily administration is also available with overlapping effectiveness [80]. With the aim of establishing CV effectiveness and safety, the Trial to Evaluate Cardiovascular and Other Long-term Outcomes with Semaglutide in Subjects with Type 2 Diabetes (SUSTAIN-6) was performed [81]. Almost 3300 patients with T2D on a standard care regimen were assigned to receive once-weekly semaglutide (0.5 mg or 1.0 mg) or placebo for 104 weeks, and of interest, over than 80% of the whole population had established CV disease. The primary composite outcome, defined as MACE, occurred in 6.6% of the semaglutide group and 8.9% in the placebo group (HR 0.74; 95% CI 0.58–0.95; *p* < 0.001 for noninferiority). Nonfatal myocardial infarction occurred in 2.9% of the patients receiving semaglutide and in 3.9% of those receiving placebo (HR 0.74; 95% CI 0.51–1.08; *p* = 0.12); nonfatal stroke occurred in 1.6% and 2.7%, respectively (HR 0.61; 95% CI 0.38–0.99; *p* = 0.04). No differences in death from CV causes were assessed between groups. Except for gastrointestinal side effects, fewer serious adverse events occurred in the semaglutide group. Worthy of attention, semaglutide was associated with a significant reduction of the incidence of revascularization (coronary and peripheral), which was included among the secondary outcomes, although mostly driven by coronary revascularization (5.0% vs. 7.6%; HR 0.65, 95% CI 0.50–0.86; *p* = 0.003). Among subgroups, about 460/3297 (14.0%) of patients presented PAD at baseline. Patients with PAD had a higher risk of MACE of about 35% regardless of treatment, compared to those not suffering from PAD. The reduction in the risk of MACE was similar in patients with and without PAD; however, the absolute benefit was greater in patients with PAD (absolute RR 4.63% points, 95% CI −0.58 to 9.84) than those without (absolute RR 1.90% points, 95% CI 0.00 to 3.80) [82]. In another multicenter, double-blind, randomized, placebo-controlled trial—the SELECT study–patients with previous CV disease, including PAD and increased body mass index, without a history of T2D, were enrolled with the aim to investigate if the weekly injection of semaglutide reduced CV events regardless of diabetes [83]. For the first time, it was documented that a drug used to treat obesity was also able to reduce CV risk; in fact, the study reached its primary target by showing a statistically significant reduction of 20% of MACE for people treated with semaglutide 2.4 mg compared to placebo, in over 17,000 patients after a median follow-up of approximately 3 years. Further studies to accurately assess the effect of semaglutide in PAD in T2D are ongoing. The SOUL trial was designed to enroll patients with T2D and established atherosclerotic CV disease and/or chronic kidney disease, with the aim to evaluate the effects of oral semaglutide versus placebo on MACE, with major adverse events on artery disease included as a secondary endpoint. At randomization, among 9650 participants, 15.7% had symptomatic PAD, thus providing relevant data in this setting of patients [84]. The STRIDE trial is a 52-week, randomized, double-blind, placebo-controlled, Phase 3b ongoing trial in which about 800 patients with T2D and early-stage PAD will be randomized 1:1 to once-weekly semaglutide 1.0 mg or placebo, both added to standard of care. The primary endpoint is the change in maximum walking distance on a constant load treadmill test from baseline to week 52. Secondary confirmatory endpoints include changes in pain-free walking distance and PAD-specific, health-related patient-reported outcomes (Vascular QoL Questionnaire-6) from baseline to week 52. The estimated date of completion of the study is scheduled for July 2024 and will provide key data to assess the effects of semaglutide on symptomatology and the course of PAD [85]. Pending these studies, semaglutide led to CV benefit in T2D, confirming its efficacy also in the setting of PAD and justifying its prescription in these patients.

#### 4.4.3. Exenatide

Exenatide is a once-weekly, injectable, extended-release formulation of an exendin-4-based GLP-1 RA approved for the treatment of T2D. The EXSCEL trial evaluated the effects of once-weekly 2 mg exenatide versus placebo on the rates of the primary composite MACE end point among patients with T2D after a median follow-up of 3.2 years [86]. Among 14,752 enrolled patients, 10,782 (73%) had previous CV disease. A primary composite outcome event occurred in 839 of 7356 patients (11.4%; 3.7 events per 100 person-years) in the exenatide group and 905 of 7396 patients (12.2%; 4.0 events per 100 person-years) in the placebo group (HR 0.91; 95% CI 0.83–1.00), with *p* < 0.001 for noninferiority but *p* = 0.06 for superiority. No differences were found between the two groups regarding the rates of CV death, fatal or nonfatal myocardial infarction, fatal or nonfatal stroke, hospitalization for heart failure, hospitalization for acute coronary syndrome, and the incidence of serious adverse events. The authors attributed the lack of CV efficacy in the trial to multiple factors, such as the shorter follow-up and the shorter duration of exposure to the trial regimen, the lower baseline glycated hemoglobin, and the higher rate of discontinuation when compared to the LEADER trial. In addition, the effect of exenatide on modifiable CV risk factors seemed modest. In a post hoc analysis, the risk of MACE and lower-extremity amputations in the PAD subgroup and the effects of exenatide versus placebo in patients with PAD matched with those without were assessed [87]. The EXSCEL included 2800 patients with PAD (19% of the trial population), who reported higher unadjusted and adjusted rates of MACE compared with patients without PAD (13.6% versus 11.4%, respectively) as well as a higher adjusted HR (adjusted HR 1.13, 95% CI 1.00–1.27; *p* = 0.047). As expected, patients with PAD had higher all-cause mortality (adjusted HR 1.38, 95% CI 1.20–1.60; *p* < 0.001) and more frequent lower-extremity amputation (adjusted HR 5.48, 95% CI 4.16–7.22; *p* < 0.001). Of importance, there were no differences between exenatide and placebo regarding rates of MACE and amputations regardless of the presence or the absence of PAD [87]. These overall data do not support a clear CV and limb net benefit by the use of exenatide in patients with T2D and PAD, despite a good safety profile and not harmful CV impact.

#### 4.4.4. Dulaglutide

Dulaglutide is a GLP-1 RA comprising two modified human GLP-1 molecules covalently linked to an IgG4 heavy chain molecule. This medication has a half-life of 5 days and is administered subcutaneously at weekly doses of 0.75 mg or 1.5 mg [88]. To assess if dulaglutide safely reduces CV risk in addition to existing antidiabetics drugs, the Researching Cardiovascular Events with a Weekly Incretin in Diabetes (REWIND) trial was designed [89]. In this multicenter, randomized, double-blind, placebo-controlled trial, men and women at least 50 years old with T2D who had a previous CV event or CV risk factors were randomly assigned (1:1) to a weekly subcutaneous injection of dulaglutide (1.5 mg) or placebo. The primary outcome was the first occurrence of the composite endpoint of nonfatal myocardial infarction, nonfatal stroke, or death from CV causes (including unknown causes), which was assessed in the intention-to-treat population. Among 9901 participants and during a median follow-up of 5.4 years, 594 (12.0%) participants at an incidence rate of 2.4 per 100 person-years in the dulaglutide group and 663 (13.4%) participants at an incidence rate of 2.7 per 100 person-years in the placebo group experienced the primary composite outcome (HR 0.88, 95% CI 0.79–0.99; *p* = 0.026). All-cause mortality did not differ between groups (10.8% in the dulaglutide group vs. 12.0% in the placebo group; HR 0.90, 95% CI 0.80–1.01; *p* = 0.067). Regarding safety concerns, gastrointestinal adverse events occurred more frequently in the dulaglutide group than in the placebo group (47.4% vs. 34.1%; *p* < 0.0001). In a post hoc analysis of the REWIND, Ferrannini and coworkers have documented that the incidence of atherosclerosis-related secondary outcomes was also lower in the dulaglutide group (HR 0.91; 95% CI 0.83–0.99; *p* = 0.03), supporting the hypothesis that dulaglutide might retard the progression of atherosclerosis [90]. To the best of the authors’ knowledge, no published studies to verify the effectiveness and safety of dulaglutide in patients with T2D and PAD are available. The current data suggest that dulaglutide improves CV profile in patients with T2D with or at risk of CV disease, probably by reducing atherosclerotic burden, thus supporting its administration also in PAD setting even though tailored research is needed.

#### 4.4.5. Albiglutide

Albiglutide is a long-acting GLP-1 RA with weekly injection, generated through genetic fusion of modified human GLP-1 to human albumin. In accordance with regulatory guidance, the HARMONY OUTCOMES trial has been conducted to assess the CV safety and efficacy of albiglutide [91]. In this double-blind, randomized, multicenter, placebo-controlled trial, patients aged 40 years and older with T2D and CV disease were assigned to groups that either received albiglutide (30 or 50 mg depending on tolerability and glycemic control) or placebo, in addition to standard care. Among 9463 participants and during a median follow-up of at least 1.6 years, the primary composite outcome—represented by the first occurrence of CV death, myocardial infarction or stroke—occurred in 7% of patients at an incidence rate of 4.6 events per 100 person-years in the albiglutide group and in 9% of patients at an incidence rate of 5.9 events per 100 person-years in the placebo group (HR 0.78, 95% CI 0.68–0.90), documenting the superiority of albiglutide vs. placebo in CV prevention (*p* < 0.0001 for noninferiority; *p* = 0.0006 for superiority). The incidence of serious adverse events did not differ between the two groups. With attention to coronary events, albiglutide administration resulted in a 25% relative risk reduction in myocardial infarction, regardless of type or ST elevation [92]. Awaiting further specific studies on peripheral atherosclerosis, actually, albiglutide should be considered safe and effective to reduce CV risk in T2D with potential broadening of the indication also to those affected by PAD, from which 25% of the participants in the trial suffered.

#### 4.4.6. Lixisenatide

The Evaluation of Lixisenatide in Acute Coronary Syndrome (ELIXA) is a multicenter, randomized, double-blind, placebo-controlled trial that addressed the question if lixisenatide, a once-daily GLP-1 RA, had CV effects in diabetic patients with recent coronary event [93]. These authors concluded that the addition of lixisenatide to usual care did not significantly affect MACE or serious adverse events. Hence, in view of this, lixisenatide should not be considered to be the first choice in T2D with PAD.

### 4.5. DPP-4 Inhibitors

DPP-4 inhibitors or gliptins belong to the class of incretin mimetics, leading to an average reduction of glycated hemoglobin of 0.5–1.0% on monotherapy but with the best glycemic effects in combination with other antidiabetics. Alogliptin, linagliptin, saxagliptin, sitagliptin, and vildagliptin are widely available and administered orally, once daily, before or after meals. Regarding the mechanism of action, DPP-4 is a ubiquitous enzyme that maintains glucose homeostasis by degrading GLP-1 and gastric inhibitory peptide (GIP). By inhibiting DPP-4, these drugs increase the levels of GLP-1 and GIP, which in turn increase insulin secretion by pancreatic beta-cells, therefore reducing postprandial and fasting hyperglycemia. The most common but also rare side effects noticed with the DPP-4 inhibitors are upper respiratory and urinary tract infections, with such reported reactions of hypersensitivity. Despite some reports, the causal relationship between DPP-4 inhibitors and pancreatitis remains unproven [94]. Besides glycemic effects, DPP-4 inhibitors exert CV activity mostly by increasing GLP-1 but also by other substrates. Stromal cell-derived factor-1α (SDF-1α), for example, is a peptide that promotes cardiac homing of endothelial progenitor cells to stimulate angiogenesis and modulate myocardial perfusion. As a substrate of DPP-4, inhibition of SDF-1α degradation preserves its effects with considerable benefit in acute myocardial infarction, cardiac recovery after ischemia-reperfusion, or stroke [95]. Moreover, DPP-4 inhibitors preserve endothelial function by increasing endothelial nitric oxide synthase phosphorylation and decreasing angiotensin II-mediated Nox-4 production, thus leading to improved endothelial function and vasodilation and reduced vascular damage and oxidative stress [95]. In line with FDA recommendations, DPP-4 inhibitors have undergone CV outcome trials to test CV safety and efficacy in T2D patients [96]. In general, DPP-4 inhibitors did not show an increased risk of CV death, nonfatal myocardial infarction, or nonfatal stroke when compared to placebo, although saxagliptin had an association with an increased rate of hospitalization for heart failure [96]. Moreover, deserving of attention are data from a retrospective propensity score-matched study on over 80,000 pairs of DPP-4 inhibitor users vs. nonusers examined for the period 2009–2011. Compared with nonusers, DPP-4 inhibitor users were associated with a lower risk of PAD (HR 0.84; 95% CI 0.80–0.88) and lower-extremity amputation (HR 0.65; 95% CI 0.54–0.79) [97]. In another real-world, retrospective head-to-head study on a total of 948,342 T2D subjects, GLP-1 RA had a lower risk of MALE (HR 0.63, 95% CI 0.41–0.96) and MACE (HR 0.62, 95% CI 0.51–0.76) when compared to DPP-4 inhibitors [98]. Similarly, further real-world data have documented that SGLT2i had a comparable risk of ischemic stroke and myocardial infarction but were associated with a relative risk reduction of 34% for congestive heart failure, 27% for lower-limb revascularization, 57% for amputations, 33% for CV deaths when compared with propensity score-matched DPP-4 group [99]. Moreover, in patients who underwent PAD revascularization, SGLT2i showed a lower risk of CV death (HR 0.60, 95% CI 0.40–0.90) but comparable risk of adverse limb events when compared with propensity score-matched DPP-4 group [100]. Taken together, these results suggest that, despite some promising data as further reported below, DPP-4 inhibitors should be considered a second choice as compared to GLP-1 RA or SGLT2i in patients with T2D and PAD.

#### 4.5.1. Alogliptin

Long-term CV safety of alogliptin is being established in a randomized, placebo-controlled clinical study in patients with T2D and acute coronary syndrome, the EXAMINE study (EXamination of cArdiovascular outcoMes with alogliptIN versus standard of carE in patients with type 2 diabetes mellitus and acute coronary syndrome) [101]. Approximately 5400 participants were recruited and followed for 4.5 years with MACE as the primary endpoint. No differences between alogliptin and placebo were found regarding CV death (4.1% vs. 4.9%, respectively; HR 0.85, 95% CI 0.66–1.10), and mortality rates after nonfatal events were comparable between groups. In the absence of specific research designed for people with PAD, deserves to be mentioned another prospective, randomized, open-label, blinded-end point, multicenter, parallel-group, comparative study—The Study of Preventive Effects of Alogliptin on Diabetic Atherosclerosis (SPEAD-A)—that addressed the question if alogliptin could attenuate progression of atherosclerosis in T2D [102]. In this study, Alogliptin was able to significantly reduce the intima–media thickness of carotid arteries than standard therapy, thus suggesting a role in preventing vascular damage [102].

#### 4.5.2. Linagliptin

To evaluate the effect of linagliptin on CV outcomes in T2D at high CV or renal risk, the CARMELINA trial was performed [103]. In this randomized, placebo-controlled, multicenter noninferiority trial, over 6990 patients were randomized to receive linagliptin 5 mg once daily or placebo added to usual care and observed for a median follow-up of 2.2 years. Rates and risk of MACE were similar between groups (HR 1.02, 95% CI 0.89–1.17; *p* < 0.001 for noninferiority) [103]. Some studies have investigated the effect of linagliptin on vascular damage, demonstrating the ability to improve endothelial microvascular function [104] and reducing arterial stiffness and markers of arterial inflammation [105], in particular in combination with SGLT-2 inhibitors [106]. These promising data encourage the use of linagliptin in subjects also affected by PAD, but targeted studies are lacking.

#### 4.5.3. Saxagliptin

In the SAVOR-TIMI 53 trial CV outcomes on saxagliptin, 16,492 patients with T2D who had a history of or were at high risk of CV events were tested [107]. Though saxagliptin did not increase or decrease the rate of ischemic events (HR 1.00; 95% CI 0.89–1.12; *p* = 0.99 for superiority; *p* < 0.001 for noninferiority in comparison with placebo), the rate of hospitalization for heart failure increased (3.5% vs. 2.8%; HR 1.27; 95% CI 1.07–1.51; *p* = 0.007). On the vascular side, Koska et al. did not find evidence that saxagliptin improves adipose tissue arteriole vasodilation or postprandial whole-body endothelial function [108]. In accordance with these data, the use of saxagliptin in patients with T2D and PAD should not be considered.

#### 4.5.4. Sitagliptin

In the TECOS trial—a randomized, double-blind study in which over 14,000 T2D patients with known CV disease were enrolled and followed for a median of 3.0 years—sitagliptin was neutral on MACE and hospitalization for angina in comparison with conventional therapy [109]. Of interest, several studies have investigated the role of sitagliptin in reducing vascular damage and atherosclerosis progression. For example, Matsubara et al. have reported that sitagliptin significantly improved endothelial function and inflammation state beyond hypoglycemic effect in a cohort of uncontrolled T2D [110]. When compared to conventional therapy, sitagliptin was able to significantly attenuate the progression of the carotid–intima–media thickness in insulin-treated T2D patients without CV history in the prospective, randomized, open-label, multicenter, SPIKE study [111]. Furthermore, as previously described, in the study published by Chang and colleagues in which DPP-4 inhibitors showed a protective effect on PAD risk, approximately 96% of participants were on sitagliptin [97]. Notably, this favorable effect was confirmed in primary prevention but not in patients with known CV disease in secondary prevention [112]. Taken together, these data support the use of sitagliptin in T2D naïve patients at high risk of—but without a history of—atherosclerotic disease such as PAD.

#### 4.5.5. Vildagliptin

To test the CV safety profile of vildagliptin, a meta-analysis of over 17,000 patients with T2D at CV risk was conducted [113]. The pooled analysis indicated that vildagliptin is not associated with an increased risk of MACE than comparators (RR 0.82, 95% CI 0.61–1.11). Referring to PAD, no available targeted data are published, but vildagliptin was able to reduce markers of vascular inflammation, lipid deposition on the vascular wall, vasoconstriction, and platelet aggregation in experimental conditions or in small clinical samples [114,115]. Conversely, vildagliptin showed a neutral effect on endothelial function and arterial stiffness, measured through peripheral artery tonometry, in patients with T2D and hypertension without known CV disease [116]. Currently, insufficient data to clearly support vildagliptin in T2D with PAD or at risk of PAD are available.

### 4.6. SGLT2i

SGLT2i or gliflozins were effective, albeit modest hypoglycemic agents when compared to other glucose-lowering drugs such as sulfonylureas or GLP-1 RA, but with substantial favorable CV/renal benefits, thus supporting their use beyond T2D [117]. In fact, SGLT2i reduces the risk of the combined endpoint of CV death and hospitalizations in patients with heart failure irrespective of ejection fraction and is recommended by both European and American guidelines for heart failure [118,119]. Going deeper into the mechanisms of action, these medications showed hypoglycemic effects by inhibiting SGLT2, which is a transporter confined to the proximal tubule of the nephron, able to modulate glucose reabsorption from urine to the bloodstream. However, a large body of evidence suggests that SGLT2i exert CV effects independently of their glucose-lowering action [120]. With a focus on atherosclerosis and vascular damage, preclinical models suggested that gliflozins improve endothelial function by increasing nitric oxide bioavailability, reducing oxidative stress and vascular inflammation, and preserving mitochondrial function [121]. Clinical trials have reported the superiority of empagliflozin, dapagliflozin, and canagliflozin vs. placebo in patients with T2D, CV risk factors, or established atherosclerotic disease, with a relative risk reduction of CV risk of 14%, mainly driven by hospitalization for heart failure [120]. Of note, several studies highlighted the CV effect of SGLT2i in the setting of PAD, in particular after the warning of the FDA and European Medicines Agency (EMA) regarding safety concerns about a potential increased risk of lower extremities amputation observed in the trials on canaglifozin. In the CANVAS (CANagliflozin cardioVascular Assessment Study) program including 10,142 patients with T2D at high CV risk, canagliflozin was associated with a 1.97-fold increased risk of lower-limb amputations, with prior amputations, male sex, non-Asian ethnicity, history of PAD, history of neuropathy, albuminuria, and increased HbA1c at baseline as independent predictors [56,122]. Furthermore, in a real-world study including 96,128 adults with CKD and T2D, newly prescribed SGLT2i were associated with a higher risk of lower-limb amputations (HR 1.65; 95% CI 1.22–2.23) and of non-vertebral fractures (HR 1.30; 95% CI 1.03–1.65) when compared with GLP-1 RA [123]. In contrast, in the CREDENCE (Canagliflozin and Renal Events in Diabetes with Established Nephropathy Clinical Evaluation) trial, including 4401 patients with T2D and CKD, similar amputation rates were found in the canagliflozin and placebo groups (HR 1.11; 95% CI 0.79–1.56) [124]. A recent meta-analysis of 20 randomized clinical trials showed that SGLT2i did not significantly change the incidence of PAD compared to conventional therapy for T2D (RR 0.98, 95% CI 0.78–1.24) [125]. In addition, subgroups analysis further revealed that the risk of incident PAD did not differ between the four evaluated SGLT2i: canagliflozin (RR 1.18; 95% CI 0.70–1.99), dapagliflozin (RR 0.86; 95% CI 0.58–1.27), empagliflozin (RR 1.16; 95% CI 0.75–1.79), and ertugliflozin (RR 0.83; 95% CI 0.49–1.40) [125]. Moreover, no increased risk of PAD or amputation with SGLT2i was observed in several studies, subanalyses, and sub-populations with different established CV diseases, as reported in detail below [126,127,128,129,130,131]. Werkman et al. have conducted a retrospective population-based cohort study on over 70,000 patients with T2D with the aim of investigating the risk of lower-limb amputations and diabetic foot ulcers with SGLT2i use and GLP-1 RA vs. sulfonylureas. SGLT2i use was not associated with a higher risk of lower-limb amputations versus sulfonylureas, while GLP-1 RA use, on the other hand, was associated with a relative risk reduction of 43% of amputations compared to sulfonylureas [65]. Of importance, the authors suggested that the difference observed between the two classes of drugs should be considered to be a powerful protective action of GLP-1 RA rather than a harmful effect of SGLT2i [65]. Regarding other competitors, no significant differences were found in the risk of lower-limb amputations between SGLT2i and DPP-4 inhibitors [132]. A recent study was conducted with the aim to reveal the association between SGLT2i and restenosis risk in 1058 patients with T2D undergoing endovascular therapy for symptomatic PAD [133]. The propensity score-matching analysis demonstrated that rates of primary patency were not different between patients treated with SGLT2i and those without at two years (72.0% versus 67.8%; *p* = 0.45). Of note, approximately 15% of the matched group assumed canagliflozin [133]. Given these conflicting findings in the PAD setting but considering the undoubted overall CV and renal benefits, it is reasonable to continue to prescribe SGLT2i but only after an adequate stratification of the risk of amputation, focusing on the presence of previous amputations, neuropathy, high HbA1c at baseline, and diabetic foot ulcers [134].

#### 4.6.1. Empagliflozin

In the Empagliflozin CV Outcome Event Trial in Type 2 Diabetes Mellitus Patients (EMPA-REG OUTCOME) trial, empagliflozin led to lower rates of MACE (HR 0.86; 95% CI 0.74–0.99; *p* = 0.04 for superiority), CV mortality (3.7% vs. 5.9%; 38% relative risk reduction), hospitalization for heart failure (2.7% and 4.1%; 35% relative risk reduction), and death from any cause (5.7% and 8.3%; 32% relative risk reduction) [54]. The difference between the two groups occurred early in the trial, and the reduction of the primary CV composite endpoint was consistent for both empagliflozin doses (10 and 25 mg). Patients in EMPA-REG OUTCOME had T2D and established CV disease, including also PAD defined as the presence of any of the following: limb angioplasty, stenting, or bypass surgery; limb or foot amputation resulting from circulatory insufficiency; evidence of significant peripheral artery stenosis (> 50% on angiography, or >50% or hemodynamically significant via noninvasive methods) in ≥1 limb; and ankle-brachial index <0.9 in ≥1 ankle [54]. Based on a subanalysis of the EMPA-REG OUTCOME trial, approximately 21% of patients had PAD at baseline, and no significant difference in lower-limb amputation risk between the two groups was found [127]. Although empagliflozin did not lead to significant improvement of three-point MACE (HR 0.84; 95% CI 0.62–1.14), it was associated with significant reduction of CV death (HR 0.57; 95% CI 0.37–0.88), all-cause mortality (HR 0.62; 95% CI 0.44–0.88), hospitalization for heart failure (HR 0.56; 95% CI 0.35–0.92), and incidence of or worsening nephropathy (HR 0.54; 95% CI 0.41–0.71). Furthermore, among patients with PAD, there was a numerically—but not statistically significant—lower risk of lower-limb amputations (5.5% with empagliflozin vs. 6.3% with placebo with an HR of 0.84 and 95% CI 0.54–1.32). Regarding safety profile, no significant differences in adverse events between patients with or without PAD were found [127]. In summary, empagliflozin maintains CV benefits also in patients with T2D affected by PAD, reducing mortality, hospitalization for heart failure, and progression of renal disease with no observed increase in the risk of amputations. The reduction in CV death with empagliflozin was so consistent, as represented by the number needed to treat 29 to prevent one event over 3 years in patients with T2D and PAD. Moreover, data from post-marketing surveillance confirmed a good safety profile of empagliflozin regarding MALE in comparison with placebo [135].

#### 4.6.2. Canagliflozin

The combined Canagliflozin Cardiovascular Assessment (CANVAS) and CANVAS-Renal (CANVAS-R) Studies demonstrated a significant reduction in the primary endpoint of MACE compared to placebo (HR 0.86; 95% CI 0.75–0.97; *p* < 0.001 for noninferiority and *p* = 0.02 for superiority), despite no significant difference in CV death [56,122]. As previously anticipated, the CANVAS program also showed an unexpectedly increased and almost doubled risk of lower-limb amputations in the canagliflozin group vs. placebo (6.3 vs. 3.4 participants per 1000 patient-years: HR 1.97; CI 1.41–2.75). The risk was similar for ischemic and infective etiologies and 100 mg and 300 mg doses. Of importance, for every clinical subgroup studied, the number of amputation events projected was smaller than the number of potential MACE avoided [56,122]. A pooled analysis of CANVAS and CREDENCE trials aimed to assess the effects of canagliflozin in patients with and without PAD. Of the overall 14,543 participants from both trials, about 22% had PAD at baseline [136]. Canagliflozin reduced MACE (HR 0.76; 95% CI 0.62–0.92) in patients with PAD, with similar relative benefits for other CV and renal outcomes in participants with or without PAD at baseline (all *p*-interaction > 0.268). No increase in the relative risk of extended MALE was observed with canagliflozin, regardless of the PAD status (*p*-interaction > 0.864). Furthermore, the absolute benefits of canagliflozin were greater in those with PAD [136]. Based on subsequent more reassuring data, the FDA and EMA removed the boxed warning to the risk of amputation with canagliflozin, but close monitoring and an adequate stratification of the baseline risk is advisable to guarantee its safe application in the setting of PAD.

#### 4.6.3. Dapagliflozin

In the Dapagliflozin on the Incidence of Cardiovascular Events-Thrombolysis In Myocardial Infarction-58 (DECLARE-TIMI 58) trial, in comparison with placebo, dapagliflozin had a lower rate of renal event (HR 0.76; 95% CI 0.67–0.87), CV death or hospitalization for heart failure (4.9% vs. 5.8%; HR 0.83; 95% CI 0.73–0.95; *p* = 0.005), mostly driven by action on heart failure (HR 0.73; 95% CI 0.61–0.88) than CV death per se (HR 0.98; 95% CI 0.82–1.17) [128]. Furthermore, no significant risk reduction of MACE (HR 0.93; 95% CI 0.84–1.03; *p* = 0.17) or overall death (HR 0.93; 95% CI 0.82–1.04) with dapagliflozin was noticed. A post hoc analysis of two Phase 3 clinical trials, DAPA-HF and DELIVER, was recently performed to aim for the safety of SGLT2i in patients with PAD who are at higher risk of amputation [126]. Among the study population, a total of 809 patients (7.4%) were affected by PAD at baseline and were more likely to have a history of smoking, hypertension, T2D, coronary heart disease, or prior stroke, but less likely to have a history of atrial fibrillation. Regarding pharmacological therapy, patients with PAD were more frequently treated with statin and antiplatelet therapy than those without PAD. As regards results, patients with PAD had a higher risk of all clinical outcomes than those without (adjusted HR 1.23, 95% CI 1.06–1.43). The benefit of dapagliflozin on the primary outcome was consistent in patients with (HR 0.71, 95% CI 0.54–0.94) and without PAD (HR 0.80, 95% CI 0.73–0.88), with no interaction between PAD and effect of treatment (*p*-interaction = 0.39) [126]. Furthermore, because patients with PAD were at high absolute risk, their absolute benefit was greater, reflected in a smaller number needed to treat (17 in patients with vs. 24 in those without PAD) [126]. Lastly, the rate of amputations was similar to that reported in other trials and substantially more common among patients with PAD (4%) than those without (0.37%), mainly due to infection rather than ischemic trigger. However, amputations were not more common with dapagliflozin than with placebo. Indeed, among patients with PAD, there were numerically fewer amputations in the dapagliflozin group [128].

#### 4.6.4. Sotagliflozin

Sotagliflozin is a dual SGLT1 and 2 inhibitor that has also been shown to have beneficial cardio–renal benefits. In The Effect of Sotagliflozin on Cardiovascular and Renal Events in Patients with Type 2 Diabetes and Moderate Renal Impairment Who Are at Cardiovascular Risk (SCORED) trial’s, the original endpoint of MACE and co-primary endpoint of CV death or hospitalization for heart failure were lower with sotagliflozin with a relative risk reduction of 16% and 23%, respectively; the final primary endpoint, defined as total number of CV death, hospitalization and urgent visits for heart failure, was significantly lower with sotagliflozin versus placebo (5.6% vs. 7.5%; HR 0.74; 95% CI 0.63–0.88; *p* < 0.001) [137]. Similar CV benefits were observed in The Effect of Sotagliflozin on Cardiovascular Events in Patients with Type 2 Diabetes Post Worsening Heart Failure (SOLOIST-WHF) trial, in which adverse events leading to amputations occurred in four versus one patient (0.7% vs. 0.2%) in the sotagliflozin and placebo groups, respectively [130]. These trials also ended early because of a loss of funding from the sponsor [130,137]. To the best of the authors’ knowledge, no targeted studies were conducted to assess the risk of PAD progression or lower-limb amputations with the use of sotagliflozin, but no differences with other SGLT2i were reported by comparative meta-analysis on safety profile [138].

#### 4.6.5. Ertugliflozin

In The Cardiovascular Outcomes Following Ertugliflozin Treatment in Patients with Type 2 Diabetes Mellitus and Atherosclerotic Cardiovascular Disease (VERTIS-CV) trial, ertugliflozin did not significantly reduce MACE or first death for composite CV/hospitalization for heart failure, but overall significantly reduced risk of first hospitalization for heart failure by 30% [139]. Amputations were performed in 2.0% of the ertugliflozin group vs. 1.6% in the placebo group [139]. Waiting for tailored research in a PAD setting, a pooled analysis of seven Phase 3 randomized controlled trials focused on the safety profile of ertugliflozin, reporting that lower-limb amputations occurred only in 0.2% and 0.5% in the ertugliflozin group (at 5 and 15 mg dose, respectively) vs. 0.1% in pooled placebo [140].

### 4.7. GIP/GLP-1 RA

A new class of drugs with a combined agonist action on both GIP and GLP-1 receptors has been developed to take advantage of this synergistic effect. Tirzepatide is the first “twincretin”, a synthetic peptide composed of 39 amino acids based on the GIP native sequence, combining the dual agonism of GIP and GLP-1 receptors and is under approval in several countries for the treatment of T2D and obesity with once-weekly subcutaneous administration [141]. In summary, the SURPASS trials have demonstrated that tirzepatide: (1) exhibited great effectiveness in improving glycemic control as monotherapy without an increased risk of clinically significant or severe hypoglycemia and leading to a significant dose-dependent reduction of body weight (7–9.5 kg) compared to placebo [142]; (2) was noninferior and superior at all doses to semaglutide with respect to the mean changes in HbA1c levels from baseline at 40 weeks (between −2.0–2.3% vs. −1.86%) [143]; (3) maintained efficacy also in combination with metformin with or without SGLT2i in comparison with insulin degludec [144]; (4) improved cardio-metabolic profile by reducing blood pressure, LDL cholesterol, and triglyceride in patients with T2D at high CV risk and slowed the rate of decline of eGFR in comparison with insulin glargine [145]. Regarding CV outcome, the expected CV benefits remain to be confirmed in the forthcoming SURPASS-CVOT, a large Phase 3, randomized, double-blind, cardiovascular outcome trial, assessing both the noninferiority and superiority of tirzepatide against dulaglutide [146]. In the meantime, Sattar and coworkers conducted a meta-analysis in which tirzepatide did not increase the risk of MACE, showing a neutral effect [147]. Moreover, in a subsequent study analyzing eight trials, Patoulias and collaborators showed that tirzepatide was associated with significantly reduced risk of MACE by 48% (RR 0.52, 95% CI 0.38–0.72), CV death by 49% (RR 0.51, 95% CI 0.29–0.89), as well as all-cause death by 49% (RR 0.51, 95% CI 0.34–0.77), compared to the control group [148]. On the other hand, some potential adverse events were described and concern nausea, vomiting, diarrhea, constipation, upper abdominal discomfort, decreased appetite, and abdominal pain [141]. Considering its promising bench-to-bedside cardio-metabolic effects, the ongoing SURPASS-CVOT trial will be important also to test the action of tirzepatide in diabetes-related lower-limb complications since 25.3% of the over 13,000 participants suffer from PAD.

### 4.8. Other Drugs

In addition to the above-described therapies, other older glucose-lowering agents are commonly used in T2D treatment. Among these, acarbose is an orally active alpha-glucosidase inhibitor with antihyperglycemic action by restricting dietary carbohydrate absorption. Regarding the CV effects of acarbose, the MEta-analysis of Risk Improvement under Acarbose (MeRIA) has analyzed the results of seven long-term trials carried out in patients with T2D. The primary endpoint was time to develop a CV event. Treatment with acarbose reduced the risk of any CV event (angina, heart failure, and stroke) by 35% [149]. In a substudy of the STOP-NIDDM trial, acarbose was able to slow the progression of vascular damage by reducing the annual increase in carotid–intima–media thickening by approximately 50% in patients with impaired glucose tolerance [150]. Despite these promising findings, no data on acarbose and PAD occurrence or progression are available; on the other hand, acarbose is not commonly utilized because of its relatively limited glucose-lowering effect and non-negligible gastrointestinal side effects. Glinides, such as repaglinide, are considered to be insulin secretagogues, thus providing improved control of postprandial glucose levels [151]. Although such studies have demonstrated a better CV profile in comparison with sulfonylureas [152], evidence of the effectiveness and safety of glinides in a PAD setting is lacking.

## 5. Discussion

PAD is a complex condition that is highly prevalent in patients with T2D, affecting prognosis, morbidity, and mortality [14,15,16,17,18,19,20]. Moreover, T2D exhibits a more severe manifestation, more distal and usually bilateral distribution of PAD, further complicating its therapeutic strategy [21,22]. Indeed, the management of PAD in patients with T2D requires a multidisciplinary and individualized approach that addresses both the systemic effects of diabetes and the specific vascular complications of PAD [134]. Adverse CV events, including MACE and MALE, represent the most important outcome in subjects affected by T2D and PAD since PAD is a well-known, often polyvascular atherosclerotic manifestation that increases the risk of lower extremities complications but also for myocardial infarction so much to be recognized as coronary disease equivalent [23,24,25,26,27,28,29]. Hence, CV prevention plays a pivotal role in this setting, encompassing smoking cessation, a healthy lifestyle modification, careful foot monitoring, and adherence to medications such as statins, antiplatelet agents, and angiotensin-converting enzyme inhibitors or angiotensin receptor blockers [153]. Alongside these drugs with established effectiveness in PAD, tailoring medical management of PAD in T2D raises considerable challenges in the choice of the best glucose-lowering medications. Notably, with the advent of novel drug classes such as GLP-1 RA and SGLT2i, the pharmacological approach to PAD in T2D might undergo—and probably already underwent—a significant watershed due to their CV benefits. Unfortunately, there are poor data obtained from targeted trials, and those available about the effectiveness and safety of antidiabetic drugs in PAD are mainly derived from post hoc analyses of subgroups or retrospective studies, thus providing few certainties or low-grade evidence [134]. Furthermore, definitions and diagnostic manner of PAD, type of outcomes, specialties involved, and follow-up period are very different among studies, leading to heterogeneous findings and fueling the complexity of the management. This review article was intended to study the literature on the topic with the aim of providing useful directions for real-life clinical practice based on a tailored perspective and an individualized approach. Since PAD dramatically increases the atherosclerotic burden and CV risk in patients with T2D beyond quality of life, the best choice of pharmacological treatment should aim to reach a pivotal endpoint: improving the risk of MALE and MACE. Of importance, in UKPDS, chronic hyperglycemia was strongly associated with both microvascular and macrovascular complications [154], while in the EUCLID, every 1% increase in HbA1c was associated with a 14% increase in the risk of MACE in patients with symptomatic PAD and T2D [155]. Hence, these findings suggest that glycemic control is fundamental in improving the CV risk of patients with PAD and T2D, and the achievement of HbA1c < 7% (<53 mmol/mol) is recommended according to international guidelines [156,157]. However, although glycemic control is important, not all glucose-lowering agents are the same in relation to CV effects. Before the breakthrough of GLP1 RA and SGLT2i, only metformin showed some CV actions with excellent safety profiles, such as improving endothelial function and reducing vascular inflammation [45,46,47], resulting in a certain protective CV effect in large studies [51,52,53,54,55,56,57]. Moreover, a significant share of participants in trials attesting to CV benefits of GLP1 RA and SGLT2i [54,55,56,77] assumed metformin at baseline, thus probably contributing to the results. With concern to PAD, both prospective and retrospective studies showed that metformin was associated with improved survival and decreased MACE and MALE [59], supporting its prescription in this setting. Regarding sulfonylureas, the strong hypoglycemic effect does not balance the discussed CV safety profile, given the direct cardiotoxicity and the high risk of hypoglycemia with questionable CV benefit, so much so that international guidelines no longer recommend this class as first-line treatment [156,157]. Then, in the era of new drugs with demonstrated CV benefit, despite some supporting and promising data, glitazones have also lost their prescribing appeal in T2D with CV disease mainly due to safety concerns about the increased risk of heart failure [71], so much so that PAD patients had no advantages in terms of CV risk with pioglitazone as reported in a subanalysis of the PROactive study [70]. DPP-4 inhibitors were shown to improve endothelial function and reduce vascular damage in experimental models [95], thus encouraging expectations about clinical settings. In patients affected by PAD, DPP-4 inhibitors reduced the risk of PAD by 16% in PAD and the risk of lower-extremity amputation by 35% in diabetic patients [97]. However, DPP-4 inhibitors showed a neutral effect on MACE in targeted trials, which is still inferior when compared to GLP-1 RA or SGLT2i in real-world studies [98,99,100]. According to these data, except for the increased risk of heart failure with saxagliptin, DPP-4 inhibitors should be considered safe in T2D with or at risk of PAD, but also a second choice when compared to GLP-1 RA or SGLT2i. Indeed, with the advent of GLP-1 RA and SGLT2i, the therapeutic approach to T2D has radically changed. Both GLP-1 RA and SGLT2i are recognized as glucose-lowering medications able to reduce the risk of MACE in patients with established CV disease or at high CV risk: more in detail, GLP-1 RA seems to have more vascular protection, thus preventing atherosclerosis progression, while SGLT2i are mostly active on hemodynamic status thus preventing hospitalization for heart failure [158]. Although the trials included patients with atherosclerosis, the population affected by PAD was underrepresented, and lower-limb complications were often not specifically considered to be primary or secondary endpoints. Regarding overall CV risk, GLP1 RA—and in particular liraglutide, semaglutide, dulaglutide, and albiglutide—were able to significantly reduce MACE in the high-CV-risk T2D population, in comparison to placebo [55,81,89]. When considering PAD, GLP-1 RA reduced MACE in both subjects with or without PAD, with absolute net benefit in the former [55,77,78,81,82,83]. Indeed, a post hoc analysis of the LEADER trial confirmed the CV benefit of liraglutide in both patients with and without PAD, with an overall reduced risk of amputations compared to placebo [77]. As also reported in the STURDUST trial, liraglutide led to improved peripheral perfusion and walking distance, thus ameliorating symptoms [78]. Of interest, in a real-world, retrospective, head-to-head study, GLP-1 RA was associated with a lower risk of MACE and MALE when compared to DPP-4 inhibitors [98]. Waiting for ongoing studies, according to current data, it is possible to affirm that GLP-1 agonists are effective and safe in PAD patients and should be considered to be the first choice in T2D with history or at high risk of PAD and limb complications. If, on the one hand, the evidence about CV and renal benefit of SGLT2i is clear and strong—in particular in patients affected by heart failure and regardless of the presence of T2D—on the other hand, data supporting their use in a PAD setting are more controversial due to an almost doubled amputation risk reported in the CANVAS trial with canagliflozin [56,122]. On the pathophysiological side, hypoperfusion to distal extremities induced by hypovolemia related to osmotic diuresis and glycosuria is the proposed and supposed mechanism underlying increased amputation risk during SGLT2i use, in a similar manner as other diuretics [159]. This safety concern was fueled by other observational studies in which SGLT2i use had an increased risk of lower-limb amputations, with most events occurring with canagliflozin [160,161]. Despite these safety concerns, neither the CANVAS–CREDENCE pooled analysis nor several other studies have reported that canagliflozin or other SGLT2i were associated with increased amputation risk. Indeed, a subanalysis of EMPA-REG on empagliflozin [127] and DAPA-HF and DELIVER [126] about dapagliflozin did not show an association between SGLT2i assumption and risk of lower-limb amputations, such as other real-life studies [131]. Based on that, the FDA and EMA removal of warnings on canagliflozin use and SGLT2i are part of the pharmacological arsenal to prevent CV risk in T2D patients. Given these conflicting findings in the PAD setting but considering the undoubted overall CV and renal benefits, it is reasonable to continue to prescribe SGLT2i but only after a better stratification of the risk of amputation, focusing on the presence of previous amputations, neuropathy, high HbA1c at baseline, and diabetic foot ulcers [134]. Despite the documented CV benefit and the availability of targeted guidelines, rates of real-life prescription of GLP-1 RA and SGLT2i in patients with T2D and PAD are suboptimal than expected, accounting for a minimum of <9% to a maximum of 22% in comparison to 75% and 56% of antiplatelets and statins, respectively [162,163,164]. These data indicate some gaps in clinical practice that this review aimed to resolve by improving knowledge and awareness about the topic. Since PAD is recognized as a complex disease, it requires a multidisciplinary approach, in which the various skills and expertise—primary care physician, diabetologist, endocrinologist, internist, cardiologist, interventional radiologist, vascular surgeon—should contribute to optimizing treatment as already shown by such studies [165]. Considering the established evidence on CV benefit, GLP-1 RA and SGLT2i should be considered to be the first choice in patients with T2D at high CV risk, alone or in combination with metformin or each other [158]. The relative magnitude of CV benefit of these drugs was recently debated in some network meta-analyses of randomized clinical trials, in which GLP-1 RA and SGLT2i showed similar efficacy in reducing atherosclerotic MACE and CV death, with trends towards more renal benefit and less hospitalization for heart failure with SGLT2i [166,167]. Regarding the PAD setting, a comparison between the two drugs in terms of lower-limb complications has demonstrated more benefit with GLP-1 RA than SGLT2i, with such an increased risk of amputations in subjects on gliflozins [161,168]. As suggested by Werkman et al., the difference observed between the two classes of drugs should be considered as a powerful protective action of GLP-1 RA rather than a harmful effect of SGLT2i [65]. In conclusion, prescription of GLP-1 RA and SGLT2i is strongly recommended to reduce overall CV risk regardless of glycemic control in T2D [142,156,157,158]; however, diabetic patients affected by PAD represent a special setting, which should be considered both the risk of MACE and MALE to choose the best therapeutic strategy in a personalized manner, preferring as first choice GLP-1 RA in those with a prevalent history or risk of lower-limb complications and SGLT2i in those with prevailing heart failure.

### Study Limitations

The main limitation of this review is the lack of studies specifically designed for the PAD setting, with data obtained and interpreted often by subanalysis of studies assessed for other aims. On the other hand, those considering people affected by T2D and PAD were often based on different definitions of PAD or lower extremities complications, duration of illness, dosages of drugs, duration of follow-up, and type of endpoints. Further investigations should be addressed on randomized head-to-head studies between glucose-lowering agents with the purpose of comparing drugs on CV outcomes in T2D and PAD settings.

## 6. Conclusions

PAD represents a common vascular complication of T2D, which leads to an increased risk of lower extremities complications and elevated overall atherosclerotic burden. In T2D and PAD settings, a multidisciplinary strategy with a tailored and personalized approach is desirable beyond mere glycemic control. These authors suggest better stratification of overall CV risk—myocardial ischemia, stroke, or CV death—and lower-limb risk—lower extremities complications and amputation—to choose the best treatment strategy for reducing MACE and MALE. Considering as monotherapy or in combination with metformin or each other, to date, GLP-1 RA and SGLT2i represent the best choice to modify CV profile in T2D patients with such clinical differences regarding PAD setting. In the case of a prevalent atherosclerotic burden with a high risk of lower-limb complications, history of previous amputations, diabetic foot ulcers, or neuropathy, these authors suggest GLP-1 RA as the best treatment. Instead, suppose the clinical context is dominated by heart failure, with clinical or instrumental evidence of fluid overload, regardless of the ejection fraction. In that case, SGLT2i should be considered a valid option if the expected overall CV benefit outweighs the risk of lower extremities complications in this setting. GLP-1 RA nevertheless represents an effective and safe therapy. Further focused research with PAD as the main endpoint and additional evidence on novel drugs, such as the dual GLP-1/GIP analogs, are needed to better clarify the complex management of PAD in patients with T2D.

## Supplementary Materials

The following supporting information can be downloaded at: https://www.mdpi.com/article/10.3390/medicina60091542/s1, Figure S1: Flow diagram of the selection process of the studies cited in the Review; Table S1: Study design, eligibility criteria, demography of study population, cardiovascular and limb outcome of the main studies considered in the Review.
