# Supplementary material for: Cardiovascular Effectiveness and Safety of Antidiabetic Drugs in Patients with Type 2 Diabetes and Peripheral Artery Disease: Systematic Review
Source: Medicina (Kaunas). 2024 Sep 20;60(9):1542. doi: 10.3390/medicina60091542 (PMC11434261; doi:10.3390/medicina60091542)
Supplement: Supplementary file 1 [file medicina-60-01542-s001.zip › medicina-3165553-supplementary.pdf]

Table S1. Study design, eligibility criteria, demography of study population, CV and limb outcome of the main studies considered in the Review

First author of the study (Ref)	Drugs and Study Description	Main Inclusion Criteria	Main Exclusion Criteria	Mean Age	Gender	Mean Follow-up	Main CV and Limb Outcome
Metformin							
Tan et al [58]	Prospective study Nov 2017-May 2018 Sample n=100<br><br>Aim: Identify the predictive factors for the onset of MALE, MACE, and death from all causes in T2D and PAD<br><br>Endpoint: MALE, MACE, death from all causes	T2D patients with PAD	N/A	N/A	N/A	12 months	Metformin was associated with a decrease of MALE (OR 0.26, 95%CI 0.10-0.68; P=0.007)
Khan et al [59]	Retrospective study Jun 2001-Dec 2014 Sample n=1,204	T2D patients with revascularization for chronic limb ischemia	T2D and PAD without revascularization	67±9 years	100% M	5 years	Metformin was associated with improved survival (HR 0.7, 95%CI 0.5-0.9; P=0.008) and decreased

	Aim: Assess the effect of metformin on outcomes after intervention for PAD<br><br>Endpoint: Primary and secondary patency, limb salvage, MALE, MACE, survival rates<br><br>Groups: Nondiabetics vs diabetics on insulin vs diabetics on metformin vs diabetics on other hypoglycemic agents						incidence of adverse cardiac events in PAD patients.<br>No impact on patency or limb salvage rates after open and endovascular interventions in comparison with insulin or other hypoglycemic agents
Kibrik et al [60]	Retrospective study<br>Oct 2012-Dec 2015<br>Sample n=187<br><br>Aim: Investigate if metformin could decrease restenosis or reintervention	T2D patients received an nDES in the superficial and/or popliteal artery	Stents placed in the iliac and tibial arteries	65±73 years	66% M	1.1 years	No statistically significant differences between groups about limb loss, restenosis, or reintervention rates

	rates in patients receiving lower extremity nDES in the superficial femoral artery and/or popliteal artery<br><br>Endpoint: Postoperative Duplex based restenosis (>60%) rates, limb loss rates, and reintervention rates<br><br>Groups: Diabetics on metformin vs diabetic on other hypoglycemic vs nondiabetics						
Sulfonylureas							
Werkman et al [65]	Retrospective study<br>2013–2018<br>Sample n=74,475<br><br>Aim: Investigate the risk of lower limb amputation and diabetic foot ulcers with SGLT2i use vs	T2D aged >18yrs at any first prescription of antidiabetic drug	Previous prescription of antidiabetic drugs	60±11 years for SGLT2-I, 56±12 years for GLP1-RA, 62±13 years for sulfonylureas , 62±12 years for DPP4 inhibitors	61%M for SGLT2-i, 55%M for GLP1-RA users, 58%M years for sulfonylureas ,	1.6 years for SGLT2-i, 3.8 years for GLP1-RA users, 3.8 years for sulfonylureas , 3.1 years for DPP4 inhibitors	Similar trends of lower limb amputations between sulfonylureas and SGLT2-I (aHR 1.10, 95%CI 0.71-1.70), but increased risk when compared with GLP1-RA

	GLP-1 RA vs DPP4 inhibitors versus sulfonylureas use<br><br>Endpoint: Incidence of lower limb amputations and diabetic foot ulcers<br><br>Groups: SGLT2i vs GLP-1 RA vs DPP4 inhibitors vs sulfonylureas				60%M for DPP4 inhibitors		(aHR 0.57, 95%CI 0.39-0.84).<br>No difference between groups about diabetic foot ulcers.
Thiazolidinediones							
Dormandy et al [70]	Post-hoc analysis of PROactive study (Randomized clinical trial)<br>May 2001-Apr 2002<br>Sample n=1274 with PAD (on a total of 5238 patients)<br><br>Aim: Comparison of CV disease outcome rates according to the	Adults aged 35–75 years with T2D and a HbA1c above the upper limit of normal.<br><br>Evidence of significant macrovascular disease, as defined by one of more of the following criteria:<br>- myocardial infarction or<br>- stroke more than 6 months before study entry;<br>- percutaneous leg intervention or coronary artery bypass graft more than 6 months before study entry;	Patients with type 1 diabetes, glucose-lowering therapy with insulin alone; NYHA class II heart failure or above; ischemic ulcers, gangrene, or rest pain in the leg; use of hemodialysis; and elevated alanine aminotransferase (>2.5 times the upper limit of normal)	63±8 years with PAD at baseline, 62±8 years without PAD at baseline	67%M with PAD at baseline, 66%M without PAD at baseline	2.5 years	There were no differences in event rates with pioglitazone versus placebo in patients with PAD at baseline, with the exception of leg revascularizations (HR 1.68, 95%CI 1.15-2.47, P=0.008). In summary, the beneficial effect of pioglitazone was seen only in patients

	presence of PAD at baseline. Evaluation of the effect of pioglitazone versus placebo in patients with PAD at baseline.<br><br>Dosages: Pioglitazone once-daily dose of 15 mg for the first month, 30 mg for the second month, and 45 mg thereafter to achieve the maximum tolerated dose within the licensed dose range.<br><br>Endpoint: Primary endpoint was MALE and MACE; the main secondary endpoint was the composite of all-cause mortality, nonfatal myocardial infarction, and stroke	- acute coronary syndrome more than 3 months before study entry;<br>- objective evidence of coronary heart disease or PAD			without PAD.

GLP-1 RA

Dhatariya et al [70]	Post-hoc analysis of LEADER trial (Randomized clinical trial) 2010-2015 Sample n=9340<br><br>Aim: Assess the impact of liraglutide versus placebo in people with T2D and at high risk of CV events on the incidence of diabetic foot ulcers and their sequelae<br><br>Dosages: Liraglutide once-daily of 1.8 mg<br><br>Endpoint: Diabetic foot ulcers, amputations, foot infections, involvement of underlying structures, or peripheral revascularization	T2D, anti-diabetic drug naïve or treated without GLP-1 analogue or DPP4, HbA1c ≥7.0%,<br><br>Prior cardiovascular disease cohort: age ≥50 and ≥1 of the following criteria:<br>-Prior MI<br>-Prior stroke or TIA<br>-Prior coronary, carotid or peripheral arterial revascularization<br>->50% stenosis of coronary, carotid, or lower extremity arteries<br>-History of symptomatic CHD documented by positive exercise stress test or any cardiac imaging or unstable angina with ECG changes<br>-Asymptomatic cardiac ischemia documented by positive nuclear imaging test, exercise test or dobutamine stress echo<br>-Chronic heart failure NYHA class II-III -Chronic renal failure: eGFR <60 mL/min (Cockcroft-Gault formula)<br><br>No Prior cardiovascular disease group: Age ≥60 y and ≥1 of the following	Type 1 diabetes ,<br><br>Calcitonin ≥50 ng/L<br><br>Use of a GLP-1 RA or pramlintide or any DPP4 inhibitor within the 3 months prior to screening<br><br>Use of insulin other than human insulin or long-acting insulin analogue or premixed insulin within 3 months prior to screening. Short-term use of other insulin during this period in connection with intercurrent illness is allowed, at Investigator's discretion<br><br>Acute decompensation of glycemic control<br><br>Acute coronary or cerebrovascular event in the previous 14 days<br><br>Currently planned coronary, carotid, or peripheral artery revascularization	64±7 years	65%M	3.8 years	Analysis of diabetic foot ulcers-related complications demonstrated a significant reduction in amputations with liraglutide versus placebo (HR 0.65, 95% CI 0.45-0.95, P = 0.03). However, no differences between placebo and liraglutide were found about the first episode of diabetic foot ulcers, foot infections, involvement of underlying structures, or peripheral revascularization in the main analysis.<br>Of importance, in the LEADER trial liraglutide was shown to reduce the occurrence of MACE both in patients with PAD (15.5% of

	Groups: Liraglutide vs placebo	criteria:<br>-Microalbuminuria or proteinuria<br>-Hypertension and left ventricular hypertrophy by ECG or imaging<br>-Left ventricular systolic or diastolic dysfunction by imaging<br>-Ankle-brachial index <0.9 acute coronary syndrome more than 3 months before study entry; or objective evidence of coronary heart disease or PAD	Chronic heart failure (NYHA class IV)<br><br>Current continuous renal replacement therapy ,<br>End-stage liver disease History of solid organ transplant or awaiting solid organ transplant<br><br>Malignant neoplasm<br><br>Family or personal history of multiple endocrine neoplasia type 2 or familial medullary thyroid carcinoma ,Personal history of non-familial medullary thyroid carcinoma				patients with liraglutide vs. 19.6% with placebo; HR without adjustment for baseline variables 0.77, 95% CI 0.58-1.01) and in those without a history of PAD (12.7% of patients with liraglutide vs. 14.1% with placebo; HR 0.89, 95% CI 0.79-1.00)
Caruso et al [78]	STARDUST trial Randomized clinical trial Feb 2021-Jun 2022 Sample n=55<br><br>Aim: Examine the effect of liraglutide on peripheral perfusion measured as peripheral transcutaneous	Age 35 years or older with type 2 diabetes and a diagnosis of PAD, evaluated with Doppler ultrasonography, computed tomography angiography, or angiography in the previous 12 months. Transcutaneous oxygen pressure (TcPO2) of the foot had to range from 49 to 30 mm Hg at the screening visit.	Previous (last 3 months) or current therapy with GLP-1RAs or dipeptidyl peptidase 4 inhibitors<br><br>Contraindications to the use of GLP-1RAs<br><br>Current or plans for pregnancy<br><br>Acute coronary and/or cerebrovascular events within the previous 14 days	68±9 years	78%M	6 months	The increase of at least 10% from baseline in TcPO2 occurred in 24 participants (89%) randomized to the liraglutide group and 13 participants (46%) assigned to the control group (relative risk, 1.91; 95% CI 1.26-2.90, P < 0.001). Moreover, TcPO2

	oxygen pressure (TcPO2) in individuals with T2D and PAD.<br><br>Dosages: Liraglutide once-daily of 1.8 mg<br><br>Endpoint: Change from baseline of (TcPo2) between groups and the comparison of the proportion of individuals who reached 10% increase of TcPO2 from baseline in each group<br><br>Groups: Liraglutide vs placebo	HbA1c ranging from 6.5% to 8%<br>Stable doses of glucose-lowering medications.	Plans or indications for peripheral revascularization procedure<br><br>Estimated glomerular filtration rate below 15 mL/min/1.73 m² <br>Neoplasms, psychiatric disorders, and use of drugs or concomitant conditions that precluded the participation in the study				increased over time in both the liraglutide group and control group with significant differences favoring the liraglutide group at the end of the trial (estimated treatment difference, 11.2 mm Hg; 95% CI, 8.0-14.5).
Verma et al [82]	Post-hoc analysis of LEADER and SUSTAIN-6 (Randomized clinical trials) Feb 2013-Mar 2016 in SUSTAIN-6 Sample n=3297 in SUSTAIN-6	For SUSTAIN-6<br>Men and women with T2D<br><br>Age ≥50 years at screening and clinical evidence of CV disease (established CV disease) or age ≥60 years at screening and subclinical evidence of CV disease	For SUSTAIN-6<br>Type 1 diabetes<br><br>Use of other GLP-1 RA or pramlintide within 90<br>days prior to screening<br><br>Use of any DPP4 inhibitors within 30 days prior to screening	For SUSTAIN-6 65±8 years	For SUSTAIN-6 61%M	For SUSTAIN-6 2.2 years	The effects of both therapies on MACE were consistently beneficial in patients with PAD (liraglutide: HR 0.77, 95% CI 0.58-1.01; semaglutide: 0.61, 0.33-1.13) and

Aim: Evaluate the CV efficacy of liraglutide and semaglutide in patients with T2D at high CV risk and PAD<br><br>Dosages: Semaglutide 0.5-1.0mg/week in SUSTAIN-6<br><br>Endpoint: MACE according to the presence of PAD at baseline<br><br>Groups: Semaglutide vs placebo in SUSTAIN-6	Antidiabetic drug naïve, or treated with one or two oral antidiabetic drug(s), or treated with human Neutral Protamine Hagedorn insulin or long-acting insulin analogue or pre-mixed insulin, both types of insulin either alone or in combination with one or two oral antidiabetic drug(s)<br><br>HbA1c ≥7.0% at screening	Treatment with insulin, other than basal and pre-mixed insulin, within 90 days prior to screening (except for short-term use)<br><br>Acute decompensation of glycemic control requiring immediate intensification of treatment to prevent acute complications of diabetes (e.g. diabetes ketoacidosis) within 90 days prior to screening<br><br>History of chronic pancreatitis or idiopathic acute pancreatitis<br><br>Acute coronary or cerebrovascular event within 90 days prior to randomization<br><br>Currently planned coronary, carotid or peripheral artery revascularization<br><br>Chronic heart failure NYHA class IV		without (liraglutide: HR 0.89, 95% CI 0.79-1.00; semaglutide: HR 0.77, 95% CI 0.58-1.01; Pinteraction = 0.34 for liraglutide and 0.49 for semaglutide). Absolute risk reductions for MACE were higher in patients with PAD (liraglutide: 4.13%-point, 95% CI -0.15-8.42; semaglutide: 4.63%-point, 95% CI -0.58-9.84) versus without (liraglutide:1.42%-point, 95% CI -0.03-2.87; semaglutide: 1.90%-point, 95% CI 0.00-3.80)

			Chronic hemodialysis or chronic peritoneal dialysis<br>End-stage liver disease<br><br>A prior solid organ transplant or awaiting solid organ transplant<br>Diagnosis of malignant neoplasm in the previous 5 years (except basal cell skin cancer or squamous cell skin cancer)<br>Personal or family history of multiple endocrine neoplasia type 2 (MEN2) or familial medullary thyroid carcinoma<br><br>Personal history of non-familial medullary thyroid carcinoma<br>Screening calcitonin ≥50 ng/l				
Badjatiya et al [87]	Post-hoc analysis of EXSCEL trial (Randomized clinical trials)<br>Jun 2010-Sep 2015<br>Sample n=2800 with PAD (on a	For EXSCEL<br>Age > 18 years<br>T2D<br>HbA1c of ≥6.5% and ≤10.0% and using one of the following treatment regimens:<br><br>Treatment with up to three (i.e. 0 – 3) oral glucose-	For EXSCEL<br>Type 1 diabetes mellitus, or a history of ketoacidosis<br>history of (≥2 episodes) of severe hypoglycemia within 12 months of enrolment	62±9 years with PAD, 62±10 years without PAD	59%M with PAD, 63%M without PAD	3.2 years	Patients treated with exenatide or placebo had similar rates of MACE and lower extremities amputations, regardless of PAD status.

total of 14,752 patients)<br><br>Aim: Assess the association of baseline PAD with rates of MACE, lower extremities amputations, and the effects of exenatide versus placebo in patients with and without PAD<br><br>Dosages: Exenatide 2mg/week<br><br>Endpoint: MACE and lower extremities amputations<br><br>Groups: Exenatide vs placebo	lowering agents (concomitant use of DPP4 inhibitors is permitted)<br><br>Insulin therapy, either alone or in combination with up to two (i.e. 0 – 2) oral glucose-lowering agents (use of basal and prandial insulins is permitted in any combination of individual or premixed insulins).<br><br>Patients with any level of CV risk and meeting all other inclusion criteria may be enrolled. Recruitment will be constrained such that approximately 30% will not have had a prior CV event and 70% will have had a prior CV event	Patient has ever been treated with an approved or investigational GLP-1 RA<br><br>Planned or anticipated revascularization procedure<br><br>Pregnancy or planned pregnancy during the trial period<br><br>Life expectancy <2 years or might limit the individual's ability to take trial treatments for the duration of the trial<br><br>End-stage renal disease or an eGFR <30mL/min/1.73m^2<br>Patient has a known allergy or intolerance to exenatide<br><br>Patient has a history of gastroparesis<br><br>Personal or family history of medullary thyroid cancer or MEN2 (Multiple Endocrine Neoplasia Type 2) or calcitonin level of >40ng/L at baseline

DPP4-i							
Chang et al [97]	Retrospective study 2009-2011 Sample n=82,169<br><br>Aim: Determine the impact of DPP-4 inhibitors on the occurrence of PAD, and lower extremity amputation risk in patients with T2D<br><br>Endpoint: PAD occurrence, amputations<br><br>Groups: DPP4 inhibitors users vs non users	T2D aged>20 years	History of peripheral arterial disease, or prescription of DPP-4 inhibitors prior to enrollment	60±12 years	54%M	3.0 years	Compared with nonusers, DPP-4 inhibitor users were associated with a lower risk of PAD (HR 0.84, 95% CI 0.80-0.88). Additionally, DPP-4 inhibitor users had a decreased risk of lower-extremity amputation than nonusers (HR 0.65; 95% CI 0.54-0.79).
Lin et al [98]	Retrospective study 2011-2017 Sample n=948,342<br><br>Aim: Determine the real-world outcomes of patients with	T2D aged>18 years without previous hypoglycemic agent prescription	Missing demographical data (<1%);<br><br>Aged <18 years;<br><br>Previously exposition to a DPP4 inhibitors or SGLT2i within 3 months before the index date;	50±13 years for GLP-1 RA, 62±13 years for DPP4 inhibitors	47%M for GLP-1 RA, 54%M for DPP4 inhibitors	2.2 years	The incidence of primary composite outcome events was significantly lower in those treated with GLP-1 RA compared with those treated with DPP4

	T2D receiving GLP-1 RA as compared with those receiving DPP4 inhibitors in terms of MACE and MALE<br><br>Endpoint: MACE, MALE<br><br>Groups: GLP-1 RA vs DPP4 inhibitors		Prior amputation				inhibitors (2.59 vs 4.22 events per 1000 person-years; subdistribution HR 0.63, 95% CI 0.41-0.96), primarily due to lower rates of amputation (1.29 events per 1000 person-years for GLP-1 RA vs 2.4 events per 1000 person-years for DPP4 inhibitors; HR 0.55, 95% CI 0.30-0.99]). Treatment with GLP-1 RA was also associated with significantly lower risks of secondary composite outcome events (11.02 vs 17.95 events per 1000 person-years; HR 0.62, 95% CI 0.51-0.76)
Lee et al [99]	Retrospective study<br>May 2016-Dec 2017<br>Sample n=11,431 on SGLT2i and	T2D with PAD aged>18 years	T2D aged<18 years	64±11 years for SGLT2i, 71±11 years for DPP4 inhibitors	50%M for SGLT2i, 48%M for DPP4 inhibitors	1 years for SGLT2i and 0.7 years for DPP4 inhibitors	SGLT2i had comparable risk for ischemic stroke and myocardial infarction, but were associated

	93,972 on DPP4 inhibitors<br><br>Aim: Evaluate the risk of CV and limb events, and death associated with the use of SGLT2i compared with DPP4 inhibitors in T2D and PAD<br><br>Endpoint: MACE, MALE<br><br>Groups: SGLT2i vs DPP4 inhibitors						with a relative risk reduction of 34% for congestive heart failure, 27% for lower limb revascularization, 57% for amputations, 33% for CV deaths, when compared with propensity score-matched DPP-4 group
Lee et al [100]	Retrospective study<br>May 2016-Dec 2019<br>Sample n=2,455 on SGLT2i and 8,695 on DPP4 inhibitors<br><br>Aim: Test the effectiveness and limb safety of SGLT2i for patients with T2D who have received revascularization for PAD	T2D with PAD aged>18 years<br><br>Revascularization for PAD	T2D aged<18 years<br><br>Previous prescription of SGLT2i or DPP4i	67±11 years for SGLT2i, 71±11 years for DPP4 inhibitors	70%M for SGLT2i, 59%M for DPP4 inhibitors	4 years	After propensity-score matching, we observed that compared with DPP4 inhibitors, SGLT2i were associated with comparable risks of ischemic stroke, acute myocardial infarction, and heart failure hospitalization but were associated with a lower risk of cardiac death (HR

	Endpoint: MACE, MALE, composite renal outcome<br><br>Groups: SGLT2i vs DPP4 inhibitors						0.60, 95% CI 0.40-0.90; P=0.0126). Regarding major limb outcomes, SGLT2i were associated with comparable risks of repeated revascularization and lower limb amputation compared with DPP4 inhibitors. SGLT2i were associated with a lower risk of composite renal outcomes (HR 0.40, 95% CI: 0.27-0.59; P<0.0001) compared with DPP4 inhibitors
SGLT2-i							
Matthews et al [56,122]	Post-hoc analysis of CANVAS and CANVAS-Renal trials (Randomized clinical trials) Dec 2009-Feb 2017 for CANVAS	Men and women with TD2;<br><br>age ≥ 30 years with history of symptomatic atherosclerotic CV disease or ≥ 50 years with two or more risk factors for CV disease	eGFR<30 mL/min/1.73 m^2 of body-surface area	63±8 years	74%M	5.7 years for CANVAS, 2.1 years for CANVAS-Renal	Rates of amputations were 6.30 and 3.37 events per 1000 partecipants-years with canagliflozin vs. placebo (HR 1.97, 95% CI 1.41-2.75) Overall amputation risk was strongly

	Gen 2014-Mar 2017 for CANVAS-Renal<br>Sample n=10,142<br><br>Aim: Assess the unexpected increased risk of major or minor lower extremity amputation<br><br>Dosages: Canagliflozin once-daily of 100-300 mg<br><br>Endpoint: lower extremities amputations<br><br>Groups: Canagliflozin vs placebo						associated with baseline history of prior amputation (major or minor) (HR 21.31, 95% CI 15.40-29.49) and other established risk factors. For every clinical subgroup studied, numbers of amputation events projected were smaller than numbers of major adverse cardiovascular events averted
Perkovic et al [124]	CREDENCE study (Randomized clinical trial)<br>Feb 2014-Oct 2018<br>Sample n=4,401<br><br>Aim: Assess the unexpected increased risk of major or minor	Man or woman ≥30 years old with a clinical diagnosis of T2D;<br><br>HbA1c ≥6.5% to ≤12.0%, (≥6.5% to ≤10.5% in Germany);<br><br>eGFR ≥30 to < 90 ml/min/1,73m^2 ;	eGFR<30 mL/min/1.73 m^2 of body-surface area	63±8 years	76%M	2.6 years	Similar amputation rates were found in the canagliflozin and placebo groups (HR 1.11, 95% CI 0.79–1.56)

	lower extremity amputation in T2D and chronic kidney disease<br><br>Dosages: Canagliflozin once-daily of 100 mg<br><br>Endpoint: lower extremities amputations<br><br>Groups: Canagliflozin vs placebo	Urinary albumin:creatinine ratio 300 mg/g to ≤5000 mg/g (>33.9 mg/mmol to ≤565.6 mg/mmol);<br><br>Stable maximum tolerated labeled daily dose of angiotensin-converting enzyme inhibitors or angiotensin receptor blockers for at least 4 weeks prior to randomization					
Verma et al. [127]	Post-hoc analysis of EMPA-REG OUTCOME trial (Randomized clinical trial) Jul 2010-Apr 2015 Sample n=1,461 with PAD at baseline (on a total of 7,020 of EMPA-REG)<br><br>Aim: Assess the risk of MACE and MALE in PAD subgroup of T2D at high CV risk	Patients adults (≥18 years of age) with: T2D;<br><br>body-mass index of 45 or less;<br><br>eGFR at least 30ml mL/min/1.73m^2 of body-surface area; established CV disease;<br><br>in the absence of glucose-lowering agents for at least 12 weeks before randomization with HbA1c level of at least 7.0% and no more than	eGFR<30 mL/min/1.73 m^2 of body-surface area	63±9 years	72%M	3.1 years	Empagliflozin led to lower rates of MACE (HR 0.86, 95% CI 0.74-0.99; P=0.04 for superiority), CV mortality (3.7% vs 5.9%; 38% relative risk reduction), hospitalization for heart failure (2.7% and 4.1%; 35% relative risk reduction), and death from any cause (5.7% and 8.3%; 32% relative risk reduction). No significant difference in

	Dosages: Empagliflozin once-daily of 10-25 mg<br><br>Endpoint: MACE, MALE<br><br>Groups: Empagliflozin vs placebo	9.0% or in therapy with stable glucose-lowering treatment for at least 12 weeks before randomization with HbA1c level of at least 7.0% and no more than 10.0%					lower limb amputation risk between the two groups was found
Butt et al. [126]	Patient-level pooled analysis of the DAPA-HF and DELIVER trials (Randomized clinical trial)<br>Feb 2017-Jul 2019 for DAPA-HF<br>Aug 2018-Mar 2022<br>Sample n=809 with PAD at baseline (on a total of 11,007)<br><br>Aim: Assess the risk of MACE and MALE in T2D and PAD subgroup<br><br>Dosages: Dapagliflozin	Patients with:<br>NYHA functional Classes II to IV;<br><br>LVEF ≤40% (DAPA-HF) or > 40% (DELIVER) and elevated N-terminal pro-B-type natriuretic peptide	History of:<br>type 1 diabetes<br><br>symptomatic hypotension or a systolic blood pressure < 95 mmHg<br>eGFR< 30 mL/min/1,73 m^2 (DAPA-HF) or < 25 mL/min/1,73 m^2 (DELIVER)	71±8 years with PAD, 70±9 years without PAD	74%M	1.8 years	The benefit of dapagliflozin on the primary outcome was consistent in patients with (HR 0.71, 95% CI 0.54–0.94) and without PAD (HR 0.80, 95% CI 0.73–0.88) ($P_{interaction}$ = 0.39). Amputations, while more frequent in PAD patients, were not more common with dapagliflozin, compared with placebo, irrespective of PAD status (PAD, placebo 4.2% vs. dapagliflozin 3.7%; no PAD,

	once-daily of 10 mg<br><br>Endpoint: MACE, MALE<br><br>Groups: Dapagliflozin vs placebo						placebo 0.4% vs. dapagliflozin 0.4%) ($P_{interaction}$ = 1.00). Infection rather than ischaemia was the main trigger for amputation, even in patients with PAD
Bhatt et al. [130]	SOLOIST-WHF trial (Randomized clinical trial) Jun 2018-Jun 2020 Sample n=1,222<br><br>Aim: Assess CV effectiveness and safety of Sotogliflozin in T2D with recent hospitalization for heart failure<br><br>Endpoint: Total number of deaths from CV causes and hospitalizations and urgent visits for heart failure	Patients with T2D, age of 18 to 85 years who were hospitalized because of the presence of signs and symptoms of heart failure and received treatment with intravenous diuretic therapy	Age < 18 or > 85 years at the Screening Visit<br><br>Index Event for worsening heart failure not caused primarily by intravascular volume overload (e.g., caused by significant arrhythmia, infection, severe anemia, or exacerbation of chronic obstructive pulmonary disease)<br><br>Hospitalization for Index Event >2 weeks<br><br>Acute coronary syndromes within 3 months prior to randomization	69±7 years	77%M	9 months	Adverse events leading to amputations occurred in four versus one patient (0.7% vs 0.2%) in the sotagliflozin and placebo groups, respectively

	(first and subsequent events)<br><br>Dosages: Sotogliflozin once-daily of 200-400 mg<br><br>Groups: Sotogliflozin vs placebo		eGFR < 30 mL/min/1.73 m^2				
Cosentino et al. [139]	VERTIS CV trial (Randomized clinical trial) Nov 2013-Dec 2019 Sample n=8,246<br><br>Aim: Assess the effect of ertugliflozin on hospitalization for heart failure and related outcomes in T2D and atherosclerotic CV disease<br><br>Endpoint: Hospitalization for heart failure, composite hospitalization for heart failure and CV death	Patients with T2D (with a glycated hemoglobin level of 7.0 to 10.5%), who were at least 40 years of age and had established atherosclerotic CV disease involving the coronary, cerebrovascular, or peripheral arterial systems	History of type 1 diabetes or ketoacidosis<br><br>eGFR below 30 mL/min/1.73m^2	64±9 years	70%M	3.5 years	Amputations were performed in 54 patients (2.0%) who received the 5 mg dose of ertugliflozin and in 57 patients (2.1%) who received the 15 mg dose, as compared with 45 patients (1.6%) who received placebo

	Dosages: Ertugliflozin once-daily of 5-15 mg<br><br>Groups: Ertugliflozin vs placebo						
Takahara et al. [133]	Prospective study<br>Mar 2018-Dec 2019<br>Sample n=1,058<br><br>Aim: Reveal the prevalence of SGLT2i treatment and its association with restenosis risk in patients with T2D undergoing endovascular therapy for symptomatic PAD<br><br>Endpoint: Primary patency<br><br>Groups: SGLT2i vs placebo	Symptomatic PAD undergoing femoropopliteal drug-coated balloon treatment in T2D	End-stage renal disease	73±8 years	67%M	1.9 years	The propensity score-matching analysis demonstrated that primary patency was not different between patients treated with a SGLT2i and those without it (72.0%, 95% CI 64.1-80.9%) versus 67.8% (95% CI 62.7-73.3%) at 2 years; P = 0.45

T2D= type 2 diabetes; PAD= peripheral artery disease; CV= cardiovascular; MALE= major adverse limb events; MACE= major adverse cardiovascular events;

N/A= not available; OR= odds ratio; HR= hazard ratio; 95% CI= confidence interval; nDES= non-drug-eluting stent; HbA1c= glycated hemoglobin;

SGLT2i= sodium-glucose transport protein 2 inhibitors; GLP-1 RA= glucagon-like peptide-1 receptor agonists; DPP-4 inhibitors= dipeptidyl peptidase 4 inhibitors;

eGFR= estimated glomerular filtration rate; TcPO2= transcutaneous oxygen pressure; NYHA= New York Heart Association; LVEF= left ventricular ejection fraction;

Figure S1. Flow diagram of the selection process of the studies cited in the Review.

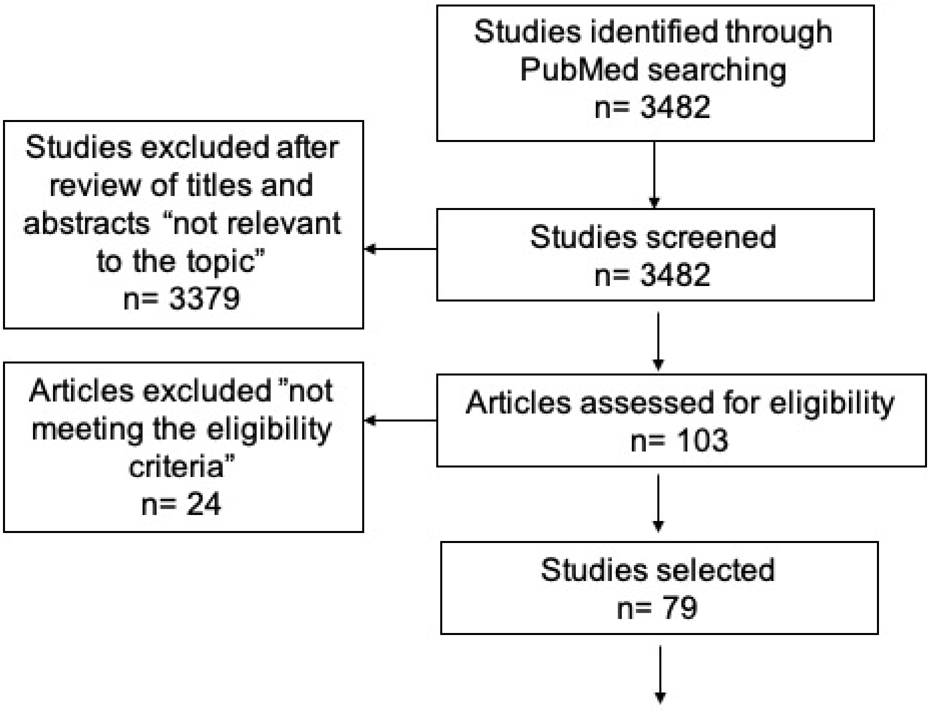

Drugs	In vitro In vivo	Overall CV outcomes in T2D	Targeted CV and limb outcomes in T2D and PAD	Ongoing studies
Metformin	3	5	3	1
Sulfonylureas	1	1	1	-
Thiazolidinediones	1	4	2	-
GLP-1 RA				
Liraglutide	2	1	2	-
Semaglutide	-	1	1	2
Exenatide	-	1	1	-
Dulaglutide	-	2	-	-
Albiglutide	-	1	-	-
Lixisenatide	-	1	-	-
DPP4 inhibitors	2	-	4	-
Alogliptin	-	2	-	-
Linagliptin	1	3	-	-
Saxagliptin	1	1	-	-
Sitagliptin	-	3	-	-
Vildagliptin	1	1	-	-
SGLT2i	2	1	6	-
Canagliflozin	-	1	2	-
Empagliflozin	-	1	2	-
Dapagliflozin	-	1	2	-
Sotogliflozin	-	1	-	-
Ertugliflozin	-	1	-	-
Acarbose	-	2	-	-
Glinides	-	1	-	-
